# Identifying mismatches between conservation area networks and vulnerable populations using spatial randomization

**DOI:** 10.1002/ece3.8270

**Published:** 2021-10-25

**Authors:** Laura A. Nunes, Christine A. Ribic, Benjamin Zuckerberg

**Affiliations:** ^1^ Department of Forest and Wildlife Ecology University of Wisconsin ‐ Madison Madison Wisconsin USA; ^2^ U.S. Geological Survey, Wisconsin Cooperative Wildlife Research Unit University of Wisconsin Madison Wisconsin USA

**Keywords:** abundance, citizen science, grassland conservation, *N*‐mixture model, spatial conservation planning, spatial simulations, species distribution models

## Abstract

Grassland birds are among the most globally threatened bird groups due to substantial degradation of native grassland habitats. However, the current network of grassland conservation areas may not be adequate for halting population declines and biodiversity loss. Here, we evaluate a network of grassland conservation areas within Wisconsin, U.S.A., that includes both large Focal Landscapes and smaller targeted conservation areas (e.g., Grassland Bird Conservation Areas, GBCAs) established within them. To date, this conservation network has lacked baseline information to assess whether the current placement of these conservation areas aligns with population hot spots of grassland‐dependent taxa. To do so, we fitted data from thousands of avian point‐count surveys collected by citizen scientists as part of Wisconsin's Breeding Bird Atlas II with multinomial *N*‐mixture models to estimate habitat–abundance relationships, develop spatially explicit predictions of abundance, and establish ecological baselines within priority conservation areas for a suite of obligate grassland songbirds. Next, we developed spatial randomization tests to evaluate the placement of this conservation network relative to randomly placed conservation networks. Overall, less than 20% of species statewide populations were found within the current grassland conservation network. Spatial tests demonstrated a high representation of this bird assemblage within the entire conservation network, but with a bias toward birds associated with moderately tallgrasses relative to those associated with shortgrasses or tallgrasses. We also found that GBCAs had higher representation at Focal Landscape rather than statewide scales. Here, we demonstrated how combining citizen science data with hierarchical modeling is a powerful tool for estimating ecological baselines and conducting large‐scale evaluations of an existing conservation network for multiple grassland birds. Our flexible spatial randomization approach offers the potential to be applied to other protected area networks and serves as a complementary tool for conservation planning efforts globally.

## Graphical abstract

Grassland birds are rapidly declining globally and therefore are of conservation concern. Here, we integrated citizen science data, hierarchical occupancy models, and novel spatial simulations to evaluate the spatial configuration of an existing conservation network for the conservation of 9 obligate grassland birds. We found representation of grassland birds within the conservation network to be high for species that specialize in moderately tall grassland habitats but not shortgrass or tallgrass specialists.

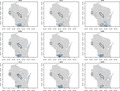

## 1 INTRODUCTION

Grasslands are one of the most endangered ecosystems in the world (Drum et al., 2015; McCracken, 2005). In North America, over 95% of tallgrass and 70–80% of mixed and shortgrass prairies have been lost due to urbanization, overt fire suppression, and agricultural intensification (Askins et al., 2007; McCracken, 2005; Wilsey et al., 2016). Concurrently, the decline of grassland habitat has contributed to population declines for many grassland birds that are uniquely adapted to native grasslands (Drum et al., 2015). As a result, grassland‐dependent birds are experiencing steeper population declines than any other avian group in North America and are of high conservation concern (Rosenberg et al., 2019; Sauer & Link, 2011).

Spatial planning for biodiversity conservation involves decisions on allocating conservation resources (e.g., optimal spatial conservation planning) and evaluating the spatial configuration of existing conservation areas to ensure that they overlap with conservation targets (Franklin et al., 2011; Stem et al., 2005). To date, these evaluations often show a low representation of conservation targets in existing conservation networks due to the lack of biological information prior to the establishment of conservation areas and the placement of conservation areas in regions with lower conflict with economic activities, regardless of biodiversity value (Jenkins et al., 2015; Xu et al., 2018).

The potential for spatial mismatches is especially high in grassland conservation networks due to persistent conflicts with agricultural activities (Thogmartin et al., 2014) and the low protection rates in grassland ecosystems relative to other major global biomes (Hoekstra et al., 2004). In addition, there is substantial variation in the structural vegetation preferences among grassland‐dependent bird species that complicate the establishment and success of broader grassland conservation efforts (Elliott & Johnson, 2017; Nocera et al., 2007; Sample & Mossman, 1997). Several grassland bird species are also area‐sensitive, whereby population density is generally higher in larger grassland patches (Murray et al., 2008; Ribic et al., 2009), and thus, one strategy for effective grassland conservation is the preservation of large, intact grasslands embedded within an agricultural landscape that provides secondary grassland habitats (e.g., pastures, hayfields; Ribic et al., 2009; Sample & Mossman, 1997; Wilsey et al., 2016).

Citizen science initiatives result in biodiversity data collection across broad geographic scales and at significantly lower costs than non‐volunteer surveys (Bonnet‐Lebrun et al., 2020; Dickinson et al., 2010; Graham et al., 2015). Structured citizen science initiatives, such as the breeding bird atlas, have advantages over “semi‐structured” initiatives (La Sorte et al., 2018) including the following: (1) systematic sampling across large spatial scales that reduces habitat biases in species estimates (Niemuth et al., 2007; Sólymos et al., 2020), and (2) standardized sampling protocols that ensure equal survey effort and also account for heterogeneous detection probabilities (Pacifici et al., 2017). These data can be useful in filling the gaps for many species and regions and therefore are extremely valuable to evaluate and inform conservation planning (Bonnet‐Lebrun et al., 2020; Bradter et al., 2018; Morán‐Ordóñez et al., 2018). Typically, biodiversity monitoring efforts were conducted using species checklists, range maps, or probability of occurrence estimates (Cantú‐Salazar & Gaston, 2013; Johnston et al., 2015). Recently, many citizen science efforts also include the collection of abundance data, which is known to improve evaluations of conservation prioritization (Johnston et al., 2015).

Here, we aimed to evaluate an existing grassland conservation network in the Midwestern State of Wisconsin, USA, a severely threatened breeding ground for North American grassland bird populations (Buxton & Benson, 2016). Wisconsin provides a breeding habitat for 17 obligate grassland bird species (Sample & Mossman, 1997), 12 of which are of greatest conservation concern (Wisconsin Department of Natural Resources, 2015). In 2010, the state initiated a landscape‐scale planning strategy to address the conservation needs of grassland‐dependent birds (Paulios, 2010). This strategy involved the identification of priority conservation areas (hereafter Focal Landscapes) that contained higher amounts of grasslands and lower amounts of suboptimal habitat (e.g., urban areas, forest) to guide the establishment of multiple “Grassland Bird Conservation Areas” within them (based on recommendations from Sample & Mossman, 1997). GBCAs are targeted landscape‐scale conservation areas and are a primary tool for the conservation of grassland birds in North America (Wilsey et al., 2016). Like other regions, these priority conservation areas were established using expert opinion and knowledge about the location of existing grasslands due to the lack of broadscale assessments of grassland bird populations (Wilsey et al., 2016). In 2019, Wisconsin completed its statewide Breeding Bird Atlas II (WBBA), which included a systematic secondary sampling scheme to measure abundance of all breeding bird species (Gibbons et al., 2007; McCabe et al., 2018). Thus, the WBBA dataset is uniquely suited for generating unbiased spatially explicit predictions of species abundance or densities for breeding bird populations for the entire state. With this dataset, it is now possible to evaluate potential mismatches between statewide grassland‐dependent bird hot spots and the placement of grassland conservation areas.

Our goal was to develop a robust baseline of the contemporary distribution and densities of a suite of obligate grassland birds and to evaluate the spatial configuration of this existing grassland conservation area for grassland‐dependent birds using a spatial randomization approach. We evaluate this spatial planning in terms of representation, in this case, the number of species with significantly high population densities, within one or multiple conservation areas (Kukkala & Moilanen, 2013). We compared the existing spatial planning against a species‐specific optimal planning strategy and a random planning strategy. We hypothesized that the representation of obligate grassland birds within the existing conservation network would be low when compared to an optimal spatial planning strategy but high compared to a random planning approach. We conducted three spatial randomizations to compare differences in representation due to the type of conservation area (i.e., Focal Landscape vs GBCAs) and spatial scale (i.e., statewide vs constrained to Focal Landscapes). In doing so, we showcase a novel framework for conducting multispecies conservation planning assessments that can be useful in monitoring other existing conservation networks and globally threatened taxa and ecosystems.

## 2 METHODS

### 2.1 Study landscapes

**FIGURE 1:**
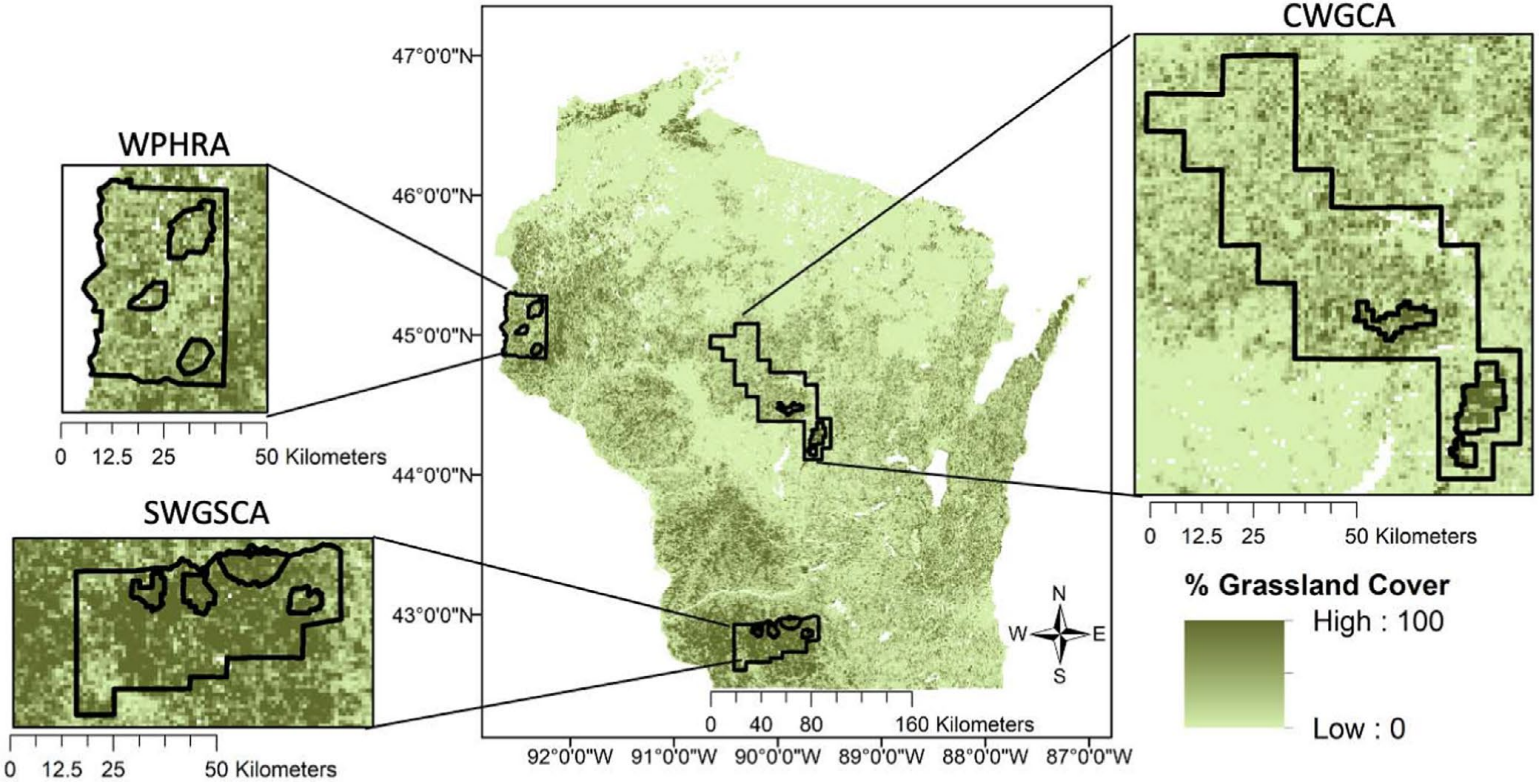
Percent of grassland cover within 1‐km^2^ grid cells in Wisconsin (Wiscland 2.0). Focal Landscapes and Grassland Bird Conservation Areas (GBCAs) overlaid. Focal Landscapes refer to areas with high amounts of grasslands (i.e., grassy matrix) and low amounts of hostile habitat (i.e., urban, forest). GBCA ensembles refer to all Grassland Bird Conservation Areas within a Focal Landscape (i.e., smaller outlines within each Focal Landscape). CWGCA, Central Wisconsin Grasslands Conservation Area; SWGSCA, Southwest Grasslands and Stream Conservation Area; WPHRA, Western Prairie Habitat Restoration Area

Wisconsin's grassland habitats are located throughout the southern and western regions of the state and are interspersed with human‐modified landscapes. In this study, we focused on three established Focal Landscapes: Southwest Grasslands and Stream Conservation Area (SWGSCA; 192,587 ha), Central Wisconsin Grasslands Conservation Area (CWGCA; 368,697 ha), and Western Prairie Habitat Restoration Area (WPHRA; 163,011 ha; Figure 1). These Focal Landscapes contain around 20% of the suitable grassland habitat in the state (i.e., grassland and agriculture land cover). These Focal Landscapes each contain multiple GBCAs (Figure 1; see Appendix S1 for details). We considered a GBCA ensemble to be the combination of individual GBCAs found within each Focal Landscape, so the GBCA ensemble of the SWGSCA Focal Landscape included four individual GBCAs, while the GBCA ensembles of CWGCA and WPHRA each contained three individual GBCAs (Figure 1). These ensembles exclude the areas within the Focal Landscape that are outside of the GBCAs. We obtained spatial datasets outlining the boundaries of these priority grassland conservation areas from Ribic et al. (2019).

### 2.2 Wisconsin Breeding Bird Atlas II

The WBBA is a structured citizen science program (La Sorte et al., 2018) that collected data on the occurrence and reproductive status of bird populations throughout the state from 2015 to 2019. General atlasing was conducted within 5 × 5 km blocks and focused on documenting species occurrences and reproductive status, with a point count‐based secondary sampling scheme implemented within each block for abundance estimation from 2016 to 2019 (McCabe et al., 2018). Observers were selected for conducting point counts using a regional bird certification test (http://www.birdercertification.org) to ensure the observers were skilled at auditory and visual observations of Wisconsin birds. Observers followed a standardized point‐count protocol in which sites were >400 m apart and surveys lasted 10 min with individual birds recorded at 0‐ to 50‐m, 50‐ to 100‐m, and >100‐m distance bins. To account for changes in detectability during surveys, observers followed a removal sampling protocol whereby only newly observed individuals at 1‐min intervals were recorded (Farnsworth et al., 2002). Surveys were conducted systematically across the state during one of the four possible years to control for potential yearly geographic biases in the sampling. In our analysis, we only included male singing detections and redetections (individuals detected as non‐singing at the start of the point count that began singing during the 10‐min period). We compiled a total of 16,542 WBBA bird point‐count sites on abundance for 10 grassland‐dependent songbirds, six of which are of conservation concern in Wisconsin (Table 1; Wisconsin Department of Natural Resources, 2015). We classified this assemblage of grassland birds based on three habitat specializations (i.e., shortgrass, midgrass, and tallgrass; Table 1). For example, Henslow's sparrows (*Centronyx henslowii*) require tall, dense herbaceous vegetation and are considered tallgrass specialists (Herkert, 2019). In contrast, horned larks (*Eremophila alpestris*) depend on short, sparse vegetation and are considered shortgrass specialists (Dinkins et al., 2019).

**TABLE 1:** Grassland‐dependent songbird species in Wisconsin and their preferences for short, intermediate, or tall vegetation heights (i.e., shortgrass, midgrass, and tallgrass, respectively; Sample & Mossman, 1997)

Species name	Scientific name	Species code	Grass height preference
Grasshopper sparrow^a^	*Ammodramus savannarum*	GRSP	Shortgrass
Horned lark	*Eremophila alpestris*	HOLA	Shortgrass
Vesper sparrow	*Pooecetes gramineus*	VESP	Shortgrass
Western meadowlark^a^	*Sturnella neglecta*	WEME	Shortgrass
Bobolink^a^	*Dolichonyx oryzivorus*	BOBO	Midgrass
Dickcissel^a^	*Spiza americana*	DICK	Midgrass
Eastern meadowlark^a^	*Sturnella magna*	EAME	Midgrass
Savannah sparrow	*Passerculus sandwichensis*	SAVS	Midgrass
Henslow's sparrow^a^	*Centronyx henslowii*	HESP	Tallgrass
Sedge wren	*Cistothorus platensis*	SEWR	Tallgrass

^a^ Wisconsin Species of Greatest Concern (Wisconsin Department of Natural Resources, 2015).

### 2.3 Hierarchical population modeling

We obtained land cover at 30‐m resolution from Wiscland 2.0 (Wisconsin Department of Natural Resources, 2016) and monthly climate data for May, June, and July (i.e., mean, minimum temperature, maximum temperature, and total precipitation) from PRISM 30‐year normals at 800‐m resolution (Daly et al., 2008). We calculated the proportion of four land cover classes (i.e., agriculture, forest, grassland, and urbanization) and the average maximum temperature and total precipitation within 100 m buffers around each WBBA point‐count site using the “landscapemetrics” R package (Hesselbarth et al., 2019); this scale approximates the maximum average territory size of grassland birds (1–3 ha) and is less than half the minimum distance between point‐count sites in this study (Sólymos et al., 2020). We found no significant multicollinearity between the selected variables (Pearson's *r* < .7). Longitude and latitude coordinates for WBBA II point‐count sites and Wisconsin's land cover and climate variables were transformed to NAD83(HARN)/Wisconsin Transverse Mercator (ESPG code: 3071).

We generated site density estimates for each species by fitting WBBA point‐count data with *N*‐mixture removal multinomial Poisson hierarchical models that account for imperfect detection (Dorazio et al., 2005; Royle, 2004). We modeled site density using the four land cover variables and two climate variables. The detection component included the annual Julian date that the surveys were conducted. We standardized all predictors by subtracting their means and dividing by the standard deviation. Models were fit with observations from the 0‐ to 50‐m distance bin to reduce observation uncertainty at longer distances (Diefenbach et al., 2003). We grouped the number of individuals of each species observed during the 10‐min observation period into 2‐min‐long discrete observation bins to use the removal method to adjust for detectability (Farnsworth et al., 2002). The fitted models also included an offset equal to the area of a circle with a 100‐m radius, which corresponds to the buffer used to measure the site‐level covariates, to estimate population density at any spatial scale. Models were fit with a maximum‐likelihood approach in the unmarked R package (Fiske & Chandler, 2011). We assessed model fit based on chi‐square goodness‐of‐fit tests and overdispersion estimates using the function *Nmix*.*gof*.*test* from the AICmodavg R package (Mazerolle, 2016). Models with overdispersion <1 were given an overdispersion value of 1. The models were corrected for overdispersion using the function *summaryOD* from the AICmodavg R package (Mazerolle, 2016). We tested for the presence of spatial autocorrelation in model residuals using the *spline*.*correlog* function in the ncf R package to calculate Moran's I between survey sites within a maximum distance of 30 km and using 100 resamples for the bootstrapping (Bjornstad & Bjornstad, 2016).

To test for the predictive accuracy of the species‐specific models in unsampled sites, we conducted a training test assessment using two approaches. First, we conducted a cross‐validation test with a 30% holdout using the CrossVal function in the unmarked R package (Fiske & Chandler, 2011). Second, we conducted an area under the curve (AUC) approach following Ball et al. (2016). In short, we fitted each species model with 70% of the data and then predicted the model onto the remaining 30% and measured sensitivity and specificity at different thresholds based on detections and non‐detections from the test dataset to measure AUC (Ball et al., 2016). We repeated this process 10 times and calculated the average AUC for each species. Species‐specific models with values higher than 0.6 were considered to have acceptable predictive accuracy (Zipkin et al., 2012). We also calculated, for each species, Spearman's rho (*r*_s_) between observed and predicted densities using the full dataset. To test for the effect of each individual site covariates on species abundances, we conducted an effect size analysis of each individual site covariate by measuring the change in abundance predictions when site covariates were increased by one standard deviation from the mean, while holding all the other covariates constant at their mean. These predictions were conducted using the modavgPred function from the AICmodavg R package (Mazerolle, 2016) while correcting for overdispersion.

To predict population densities at a meaningful spatial scale for conservation planning (Sólymos et al., 2020), the state of Wisconsin was divided into 1‐km^2^ pixels, resulting in a total of 134,295 1‐km^2^ pixels. The proportion of the same four land cover categories and mean climate within each pixel were measured and fitted using the hierarchical model estimates to generate spatially explicit predictions of population density for each 1‐km^2^ pixel in Wisconsin (see Appendix S2 for details). We predicted population densities using the average coefficients, and to account for error propagation, we generated predictions using ±1.96*SE of the model coefficients and conducted the analysis outlined below using the three sets of estimates (i.e., average, 2.5%, and 97.5% confidence intervals).

### 2.4 Spatial planning evaluations

To estimate the optimal spatial planning for each species, we identified the smallest set of 1 × 1 km grid cells, which together encompassed 20% of the statewide population of each individual species (Johnston et al., 2020). We selected a 20% conservation target so that it would be higher than the 10% threshold for critically endangered species listed by the International Union for the Conservation of Nature (IUCN). To compare the species‐specific optimal spatial designs with the existing spatial design for grassland conservation, we also measured the proportion of the statewide population that is predicted within the three existing Focal Landscapes to test how this spatial design was compared with 20% and 10% conservation thresholds.

We estimated contemporary population baselines based on the mean density estimates of all 1‐km^2^ pixels within each individual conservation area (i.e., Focal Landscape or GBCA) and within the entire state. We used a 500‐m buffer around the landscape boundaries to ensure that all pixels intersecting with the landscape boundaries were included.

We assessed the existing conservation network for obligate grassland songbirds in terms of species representation using a spatial randomization, or null model, approach (Gotelli, 2001; Thornton et al., 2016). We defined representation of each conservation area to be the number of species with significantly higher population densities within a conservation area relative to a random distribution of replicate conservation areas. This randomization approach allowed us to compare the population baselines represented by the existing conservation network against a conservation planning approach that is not constrained by socioeconomic conflicts in predominantly grassland or agriculture landscapes. We adapted the random translation–rotation (RTR) model of Nunes and Pearson (2017) to randomly place replicate landscapes of the existing conservation areas (i.e., individual Focal Landscapes or GBCAs), thus maintaining the same size and shape of the conservation areas of interest. This approach generated a distribution of 1000 randomly placed replicate conservation areas across a sampling region (i.e., the state of Wisconsin or within a Focal Landscape). We constrained the placement of replicates to ensure replicates did not fall in predominantly unsuitable areas for grassland bird conservation (e.g., areas with predominant forest or urban land cover). To constrain the random placement of replicates to suitable grassland areas, we used a three‐step process: (1) We defined any 1‐km^2^ grid cell that had at least 50% of total unsuitable land cover (i.e., forest and urban land types) as unsuitable for grassland bird conservation; (2) we calculated the average amount of unsuitable grassland habitat within all conservation areas studied (i.e., Focal Landscapes and GBCA ensembles) based on Wiscland 2.0 (see above); and (3) we discarded any null replicates that encompassed more than 20% of unsuitable grassland bird habitat (average amount of unsuitable habitat among the studied conservation areas) and replaced them with replicates that fell in areas with <20% of unsuitable habitat, thus ensuring comparability with current conservation areas (replicates included in the analysis are found in Appendix S4).

Finally, we calculated the mean population density of each replicate landscape using the same approach as the population baselines in the existing conservation areas. We defined an existing conservation area of interest (i.e., Focal Landscapes or GBCA ensemble) to have representation for a given species when the corresponding population baseline density was greater than the 90% confidence interval of the densities generated from the randomly placed replicate areas (Nunes & Pearson, 2017; Thornton et al., 2016; see Appendix S4 for details), thus indicating that densities were significantly higher within an existing conservation area than expected by random chance. We conducted these spatial randomization tests under three different scenarios.

**FIGURE 2:**
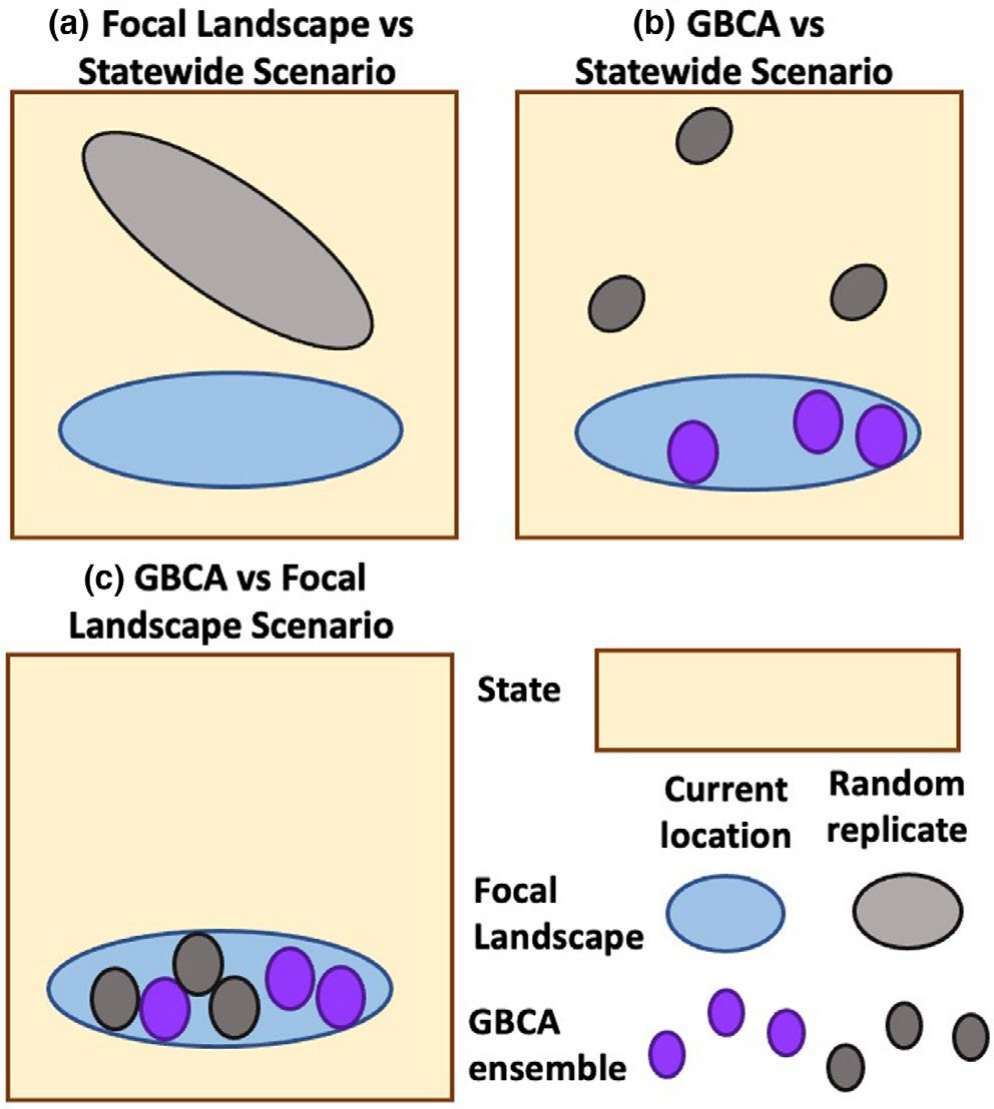
Schematic of three simulation scenarios to assess the placement of current conservation areas for grassland songbirds. (a) Population baselines in Focal Landscapes are tested against replicate landscapes randomly placed across suitable grassland habitats in the state (“Focal Landscape vs statewide” scenario); (b) population baselines in current GBCA ensembles within Focal Landscapes are tested against GBCA ensembles placed randomly across suitable grassland habitats in the state (“GBCA vs statewide” scenario); and (c) population baselines in GBCA ensembles are tested against random replicate ensembles in suitable grassland areas within their associated Focal Landscapes (“GBCA vs Focal Landscape” scenario). Focal Landscapes refer to areas with high amounts of grasslands (i.e., grassy matrix) and low amounts of hostile habitat (i.e., urban, forest). GBCA ensembles refer to all Grassland Bird Conservation Areas within a Focal Landscape

*“Focal Landscape vs statewide” scenario: Do existing Focal Landscapes overlap with higher densities of grassland songbirds relative to other suitable grassland areas in the state?* We tested whether population densities in each Focal Landscape were significantly higher relative to randomly placed replicates in suitable grassland areas in the state (Figure 2a). In doing so, we predicted that the Focal Landscapes would have representation for several grassland songbirds than randomly placed areas that are similar in amount of suitable habitat.

*“GBCA vs statewide” scenario: Do GBCA ensembles within Focal Landscapes overlap with higher densities of grassland songbirds relative to other suitable grassland areas across the state?* We tested whether the ensemble of GBCAs embedded within the Focal Landscapes was associated with higher population densities for grassland songbirds relative to replicate GBCAs placed randomly within suitable grassland areas in the state. To do this, we randomly placed individual GBCAs of each Focal Landscape in suitable grassland habitats across the state, thus simulating GBCA ensembles unconstrained by Focal Landscape boundaries (Figure 2b). Individual GBCAs were not allowed to overlap with one another to ensure that the replicates GBCA ensembles were made up of disjunct conservation areas. We predicted that all existing GBCA ensembles would have significantly higher population densities for more grassland bird species than these randomly placed GBCAs.

*“GBCA vs Focal Landscape” scenario: Do GBCAs within Focal Landscapes overlap with higher population densities of grassland songbirds relative to other suitable grassland areas across the Focal Landscape?* Using the same approach as the “GBCA vs statewide” scenario, we constrained the sampling region to the current extents of the corresponding Focal Landscape (Figure 2c). We predicted that the current location of all GBCAs would be associated with higher population densities for more grassland songbirds than other randomly placed GBCAs within the Focal Landscapes.

All analyses described above were developed and conducted in R (R Core Team, 2020).

## 3 RESULTS

### 3.1 Model results

Of the 10 species studied, the most detected species was Savannah sparrow (1014 individuals at 814 sites), and the least commonly detected species were Henslow's sparrow (26 individuals at 22 sites) and western meadowlark (12 individuals at 12 sites). Bobolink, dickcissel, and western meadowlark were the only species that had a low model fit (chi‐square *p*‐value of <.05). Most models had overdispersion estimates between 1 and 2, except for dickcissel, Henslow's sparrow, and western meadowlark. Spline correlograms showed Moran's *I* estimates centered around zero, with quantiles overlapping with zero, therefore indicating a lack of residual spatial autocorrelation (Dormann et al., 2007). Spearman's rho (*r*_s_) was positive between observed and predicted densities for all species (*r*_s_ = 0.03 to 0.3) with the lowest correlations for species observed the least (grasshopper sparrow (*r*_s_ = 0.07); Henslow's sparrow (*r*_s_ = 0.05); and western meadowlark (*r*_s_ = 0.03)). Lower correlations in datasets with large numbers of zeros (such as these rarer species) result from predicted densities having an adjustment for detectability, which binds values away from zero. AUC scores ranged from 0.69 to 0.9, except for the western meadowlark (AUC = 0.4), and thus, the western meadowlark was excluded from the rest of the analysis due to low predictive accuracy. As expected, population densities tended to increase with increasing grassland or agriculture land cover and decrease with increasing urban or forest areas, but these relationships varied across species (see Appendix S2 for full details on model results and assessment).

### 3.2 Spatial density patterns

**FIGURE 3:**
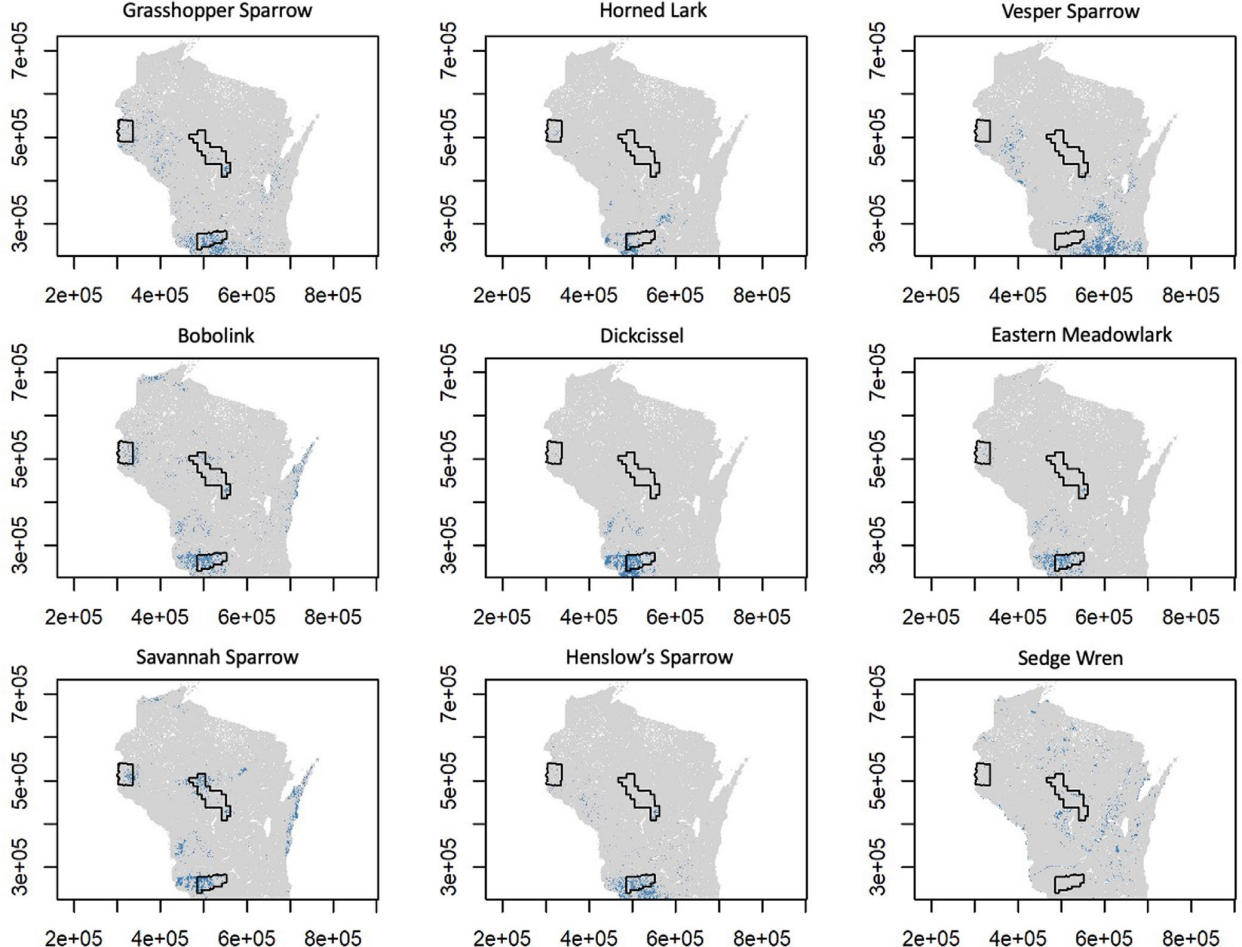
Optimal spatial planning for nine obligate grassland songbirds grouped by habitat specialism (blue for shortgrass specialists, purple for midgrass specialists, and green for tallgrass specialists). Blue pixels represent the smallest set of 1‐km^2^ grid cells that were predicted to encompass at least 20% of the statewide population. Outlines of existing Focal Landscapes are overlaid in black

**FIGURE 4:**
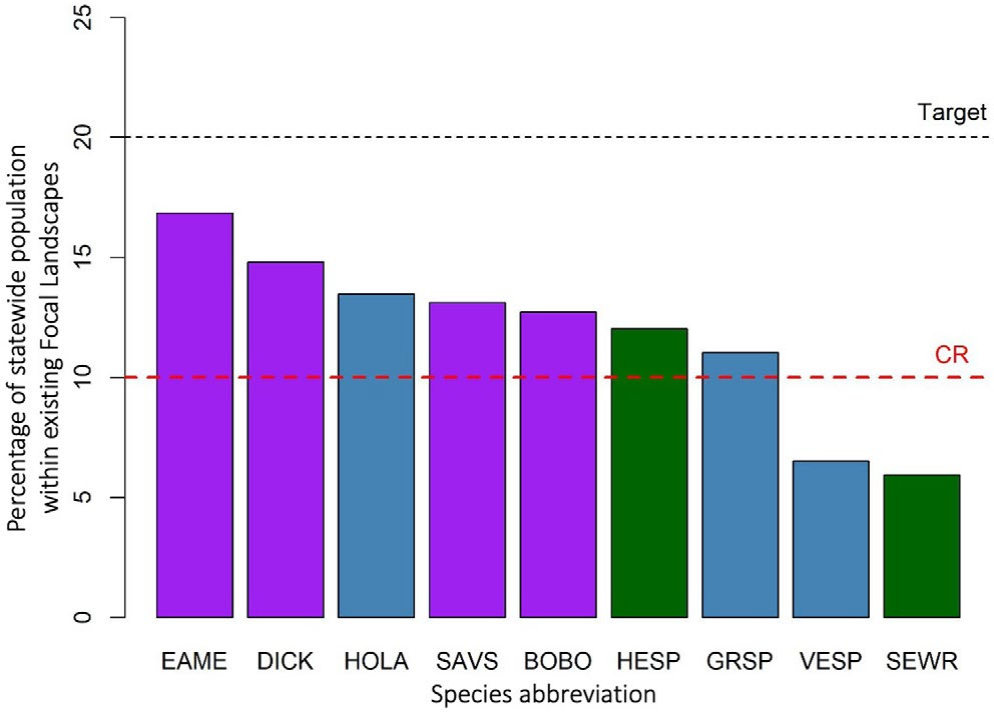
Percentage of statewide population within existing Focal Landscapes. Colors distinguish between habitat specialists (blue for shortgrass specialists, purple for midgrass specialists, and green for tallgrass specialists). Refer to Table 1 for species abbreviations. Dotted black line represents the conservation target of 20% of statewide population to be within existing conservation areas (i.e., Focal Landscapes). Dotted red line represents the 10% threshold for IUCN’s critically endangered status (Johnston et al., 2020)

We found variation among the density patterns of the nine obligate grassland songbirds, but Southwest Wisconsin appeared to be an area with the highest density for most of the species (Figure 3). The minimum sets of 1 × 1 km grid cells that include 20% of the statewide populations for all species studied were smaller than the size of the existing conservation network (i.e., all three Focal Landscapes combined; full results in Appendix S3). Furthermore, the existing conservation network did not meet the target of protecting 20% statewide population for any of the species studied (Figure 4). The species with the highest population percentage within the conservation network were eastern meadowlark and dickcissel (midgrass specialists) and horned lark (shortgrass specialist; Figure 4). In contrast, less than 10% of statewide populations were within the conservation network for vesper sparrow (shortgrass specialist) and sedge wren (tallgrass specialist; Figure 4).

### 3.3 Contemporary population baselines

Across all species, mean population densities in the Focal Landscapes were similar to or higher than statewide average estimates (Table 2). The SWGSCA Focal Landscape had consistently higher population densities compared with the other two Focal Landscapes; the CWGCA Focal Landscape had equal to or lower population densities than the WPHRA (Table 2; see Appendices S5–S7 for details). These contemporary population baselines could be used to inform future conservation management success.

**TABLE 2:** Mean densities per km^2^ for each grassland bird species within Focal Landscapes and the state based on model coefficient averages (see Appendices S5–S6 for further details)

Species name	Scientific name	SWGSCA	CWGCA	WPHRA	State
		Sample size = 1945 × 1 km^2^	Sample size = 3670 × 1 km^2^	Sample size = 1631 × 1 km^2^	Sample size = 140,780 × 1 km^2^
Grasshopper sparrow	*Ammodramus savannarum*	0.22^a^	0.08^a^	0.10^a^	0.06
Horned lark	*Eremophila alpestris*	2.59^a^	0.63^a^	1.15^a^	0.48
Vesper sparrow	*Pooecetes gramineus*	0.37^a^	0.26^a^	0.34^a^	0.24
Bobolink	*Dolichonyx oryzivorus*	1.27^a^	0.55^a^	0.59^a^	0.30
Dickcissel	*Spiza americana*	3.96^a^	0.93^a^	1.46^a^	0.64
Eastern meadowlark	*Sturnella magna*	1.37^a^	0.24^a^	0.38^a^	0.17
Savannah sparrow	*Passerculus sandwichensis*	4.76^a^	2.59^a^	2.80^a^	1.25
Henslow's sparrow	*Centronyx henslowii*	0.18^a^	0.03	0.05^a^	0.03
Sedge wren	*Cistothorus platensis*	0.74^a^	0.62^a^	0.48	0.53
	Mean	1.71^a^	0.60^a^	0.74^a^	0.37

^a^ Mean densities that are higher in a given Focal Landscape than the state.

**FIGURE 5:**
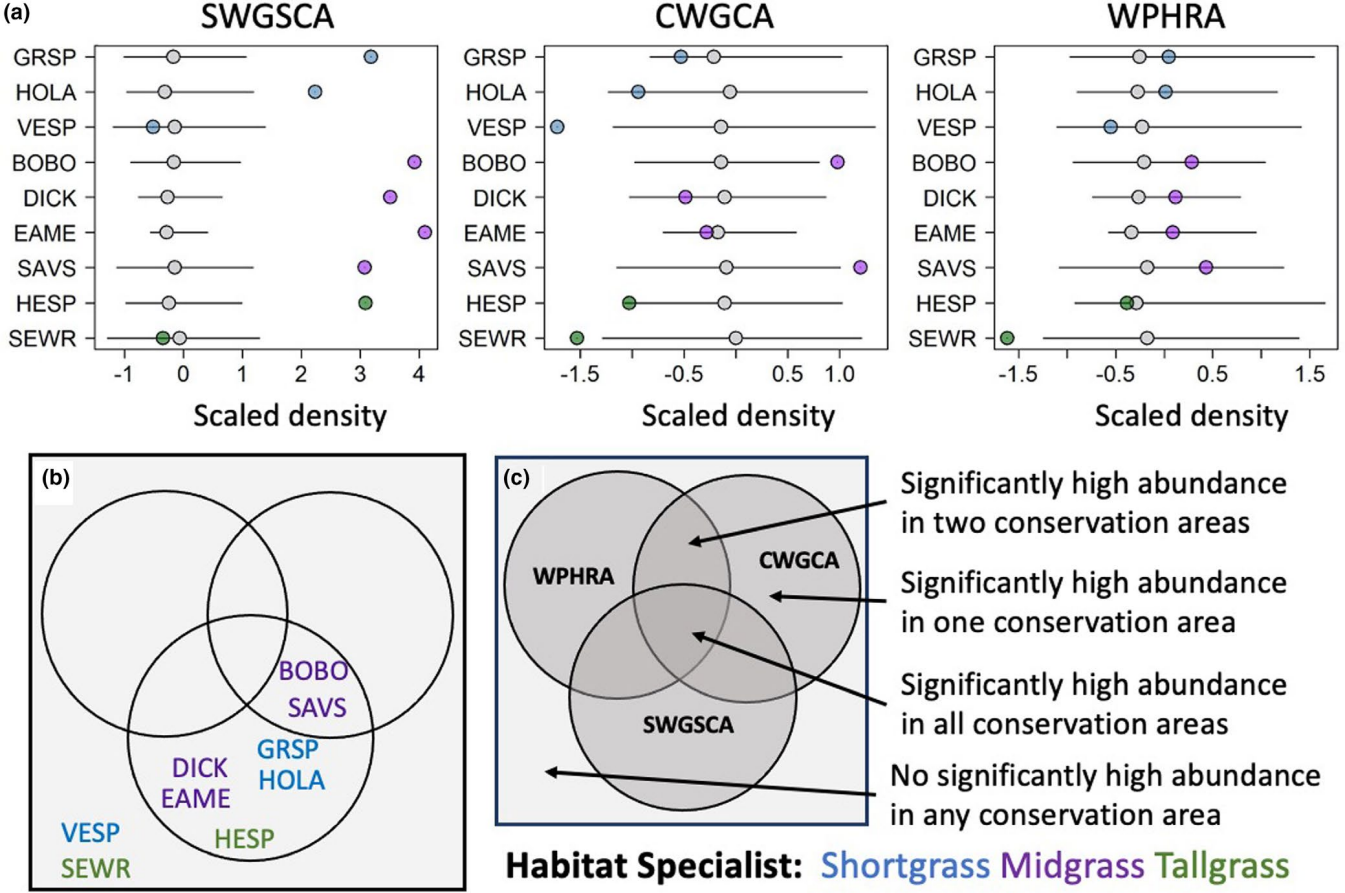
Spatial simulation testing for significantly higher population densities within existing Focal Landscapes relative to random placement in suitable grassland habitats across the state. (a) Scaled population densities from individual Focal Landscapes against null replicates for each species. Gray circles represent 50% quantile, and the black lines represent 10–90% quantiles. Cases with colored circles outside of solid lines represent population densities in existing Focal Landscapes that are significantly higher than random. (b) Diagram representing significant and nonsignificant cases for each Focal Landscape. (c) Diagram legend indicating the order of the circles as being WPHRA in the top left circle, CWGCA in the top right circle, and SWGSCA in the bottom. Significant cases refer to species where their estimated population densities within Focal Landscapes fell within the top 10% of the null distribution of replicate population densities. Colors represent the grassland habitat of each species. CWGCA, Central Wisconsin Grasslands Conservation Area; SWGSCA, Southwest Grasslands and Stream Conservation Area; and WPHRA, Western Prairie Habitat Restoration Area. Refer to Table 1 for species abbreviations

**FIGURE 6:**
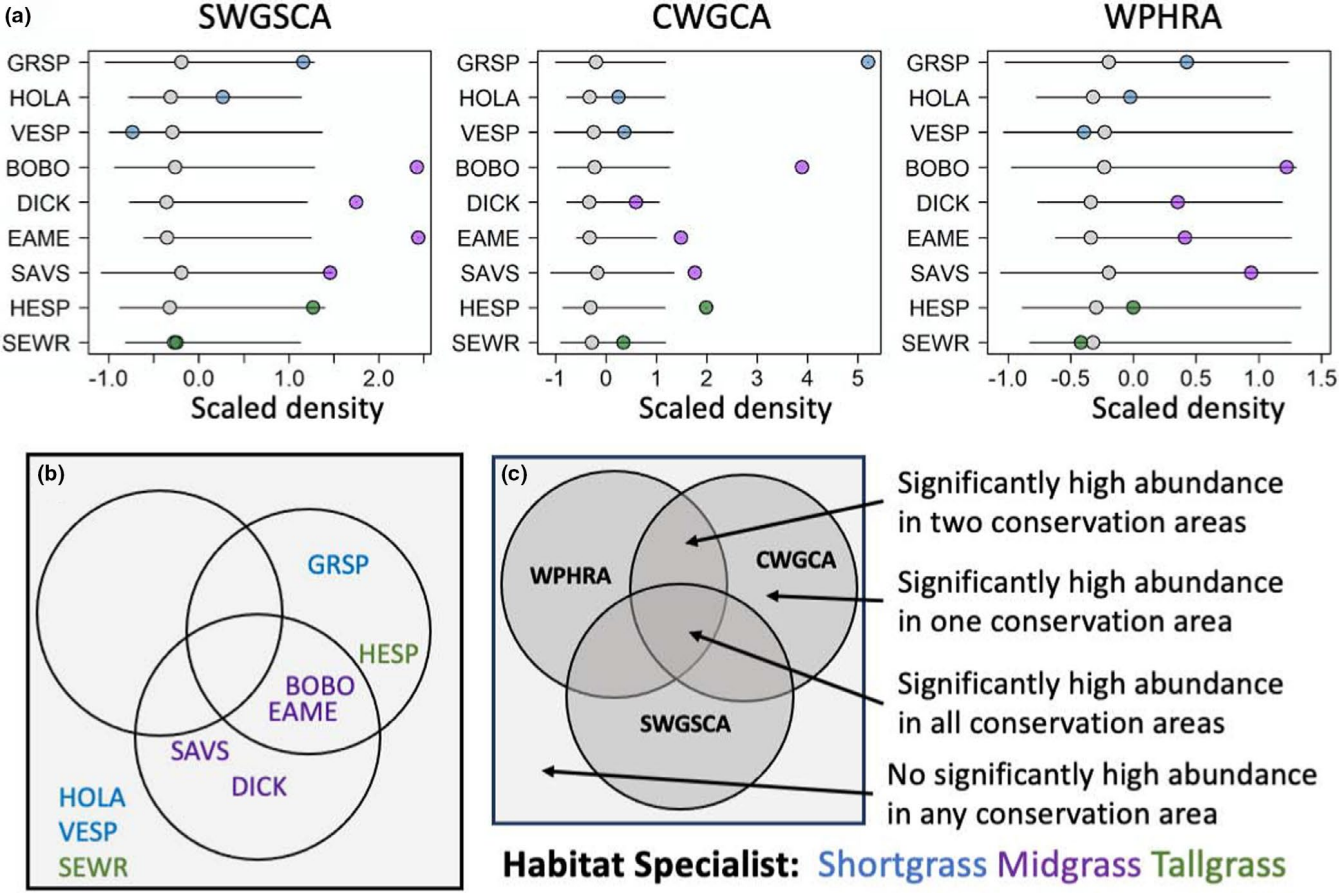
Spatial simulation testing for higher grassland bird densities within existing GBCA ensembles relative to random placement in suitable grassland habitats across the state. (a) Scaled population densities from individual GBCA ensembles against null replicates. Gray circles represent 50% quantile, and the black lines represent 10–90% quantiles. Cases with colored circles fall outside of solid lines represent population densities in existing GBCA ensembles that are significantly higher than random. (b) Diagram representing significant and nonsignificant cases for each GBCA ensemble. (c) Diagram legend indicating the order of the circles as being WPHRA in the top left circle, CWGCA in the top right circle, and SWGSCA in the bottom. Significant cases refer to species with estimated population densities within GBCA ensembles that fell within the top 10% of the null distribution of population densities. Colors represent the grassland habitat of each species. CWGCA, Central Wisconsin Grasslands Conservation Area; SWGSCA, Southwest Grasslands and Stream Conservation Area; WPHRA, Western Prairie Habitat Restoration Area. Refer to Table 1 for species abbreviations

In the “Focal Landscape vs statewide” scenario, the Focal Landscape with the highest representation of grassland‐dependent birds was SWGSCA (seven out of nine species), followed by the CWGCA (two out of nine species). The WPHRA had no species with higher densities in the focal landscape relative to random replicates (Figure 5). We also found two species (vesper sparrow and sedge wren) that were not represented in any of the Focal Landscapes (Figure 5). We found a similar pattern of representation in the “GBCA vs statewide” scenario, with the GBCA ensemble of SWGSCA having the highest representation values (four out of nine species), followed by the GBCA ensembles of CWGCA (four out of nine species). We also found three species (horned lark, vesper sparrow, and sedge wren) without higher densities between any current GBCA ensembles and GBCA replicates outside Focal Landscapes (Figure 6). Species representation between these two types of conservation areas (i.e., Focal Landscapes and GBCAs) was similar relative to statewide replicates, with the exception of horned lark not being represented in the “GBCA vs statewide” scenario.

**FIGURE 7:**
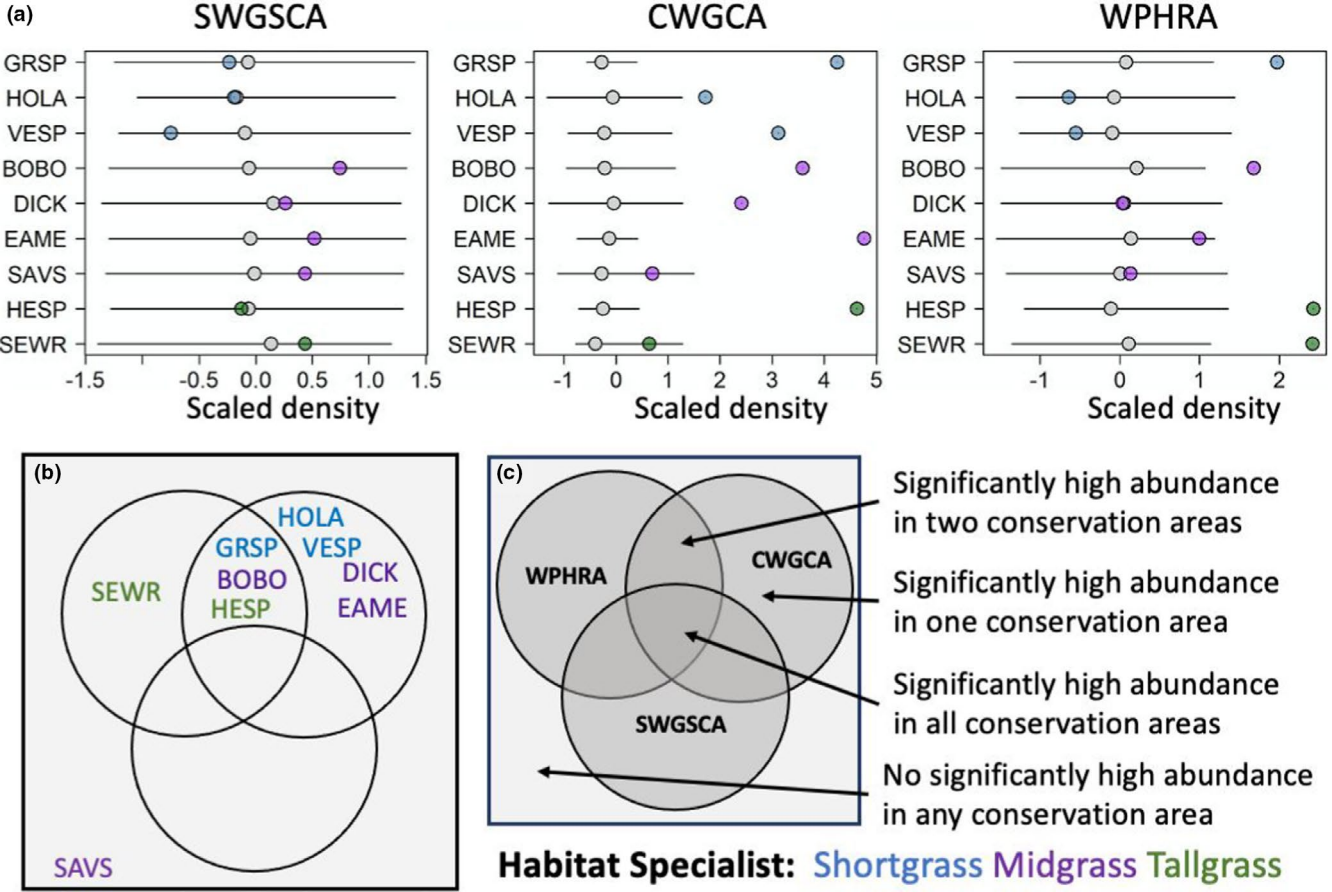
Spatial simulation testing for higher grassland bird population densities within current GBCA ensembles relative to random placement in suitable grassland habitats within the focal landscape. (a) Scaled population densities from individual GBCA ensembles against null replicates. Gray circles represent 50% quantile, and the black lines represent 10–90% quantiles. Cases with colored circles fall outside of solid lines represent population densities in existing GBCA ensembles that are significantly higher than random. (b) Diagram representing significant and nonsignificant cases for each GBCA ensemble. (c) Diagram legend indicating the order of the circles as being WPHRA in the top left circle, CWGCA in the top right circle, and SWGSCA in the bottom. Significant cases refer to species with estimated population densities within GBCA ensembles that fell within the top 10% of the null distribution of population densities. Colors represent the grassland habitat of each species. CWGCA, Central Wisconsin Grasslands Conservation Area; SWGSCA, Southwest Grasslands and Stream Conservation Area; and WPHRA, Western Prairie Habitat Restoration Area. Refer to Table 1 for species abbreviations

In the “GBCA vs Focal Landscape” scenario, the GBCA ensemble in the CWGCA Focal Landscape had the highest representation of species (seven out of nine) relative to replicate GBCAs within the Focal Landscape, followed by the WPHRA (four out of nine species; Figure 7). Under this scenario, we did not find any species having higher representation within the GBCAs of the SWGSCA Focal Landscape. Finally, Savannah sparrow did not have significantly higher densities in any GBCA ensemble compared with the Focal Landscape (Figure 7). Species representation was higher in the “GBCA vs Focal Landscape” scenario (eight out of nine species) than in the “GBCA vs statewide” scenario (six out of nine species). Across all scenarios, the midgrass specialists tended to be the most represented by this conservation network than shortgrass or tallgrass specialists (Figures 5, 6, 7).

## 4 DISCUSSION

Mismatches between the placement of conservation areas and biodiversity hot spots often result from a lack of explicit information on abundance or demographic rates when establishing protected areas (Johnson et al., 2010) and competing interests from other conservation or land use priorities (Jenkins et al., 2015). Here, we used a statewide citizen science effort to develop a spatial assessment of an existing grassland conservation network and evaluated the representation of bird populations within strategically placed grassland conservation areas. These findings provide a “snapshot” of the representation of grassland bird communities in these priority areas relative to the broader jurisdictional scales over which conservation and land acquisition decisions are made. In doing so, we compared the representation of obligate grassland songbirds within this conservation network with both optimal and random spatial planning approaches. Overall, we found the existing conservation network to have a low representation when compared to optimal conservation targets. Specifically, no species met the target of 20% statewide population protected within the existing conservation network, despite the size of this network being larger than the minimum area required to meet this target under an optimal planning approach. When the existing conservation network was compared to three randomization scenarios, we found it to have representation for more than half of the species studied. However, representation varied across scenarios, conservation areas, and species, thus highlighting the importance of conducting conservation assessments that consider multiple species and scales.

Anthropogenic disturbances disproportionately impact habitat specialists relative to habitat generalists (Keinath et al., 2017). Consequently, there is a need to implement targeted conservation to meet the distinct habitat requirements of species assemblages. The establishment of GBCAs is considered an essential tool for the conservation of grassland obligate birds in North America (Johnson et al., 2010), and in Wisconsin, the identification of Focal Landscapes served as a guide for the spatial prioritization of GBCAs. We found that this hierarchical approach results in the representation of seven out of the nine species studied in multiple GBCA ensembles, which could be a reflection of the ability of some of these species to use both agricultural and non‐agricultural grasslands (Guttery et al., 2017; Nocera et al., 2007). The higher species representation in the “GBCAs vs statewide” scenario compared with the “Focal Landscapes vs statewide” scenario highlights the importance of targeted initiatives for the conservation of obligate grassland birds relative to broadly targeted grassland management. Other strategic approaches could be adopted for species that are currently underrepresented within this hierarchical approach. Importantly, GBCAs aligned with density hot spots for all but Savannah sparrow when evaluated within smaller spatial scales (i.e., Focal Landscapes). These findings suggest that the degree of obligate grassland bird representation within GBCAs depends on the spatial extent (i.e., statewide vs. within Focal Landscapes), and highlights the importance of landscape‐scale simulations across multiple species and scales for conservation planning assessments.

In many cases, the establishment of grassland conservation areas often focuses on the protection of a single species or group of species (e.g., waterfowl, Grant et al., 2017). These umbrella species serve as surrogates for the conservation of other grassland‐dependent species, but the habitat requirements of umbrella species could make them an unsuitable surrogate for grassland‐dependent songbirds (Grant et al., 2017; McNew et al., 2015). Differences in land management goals among Focal Landscape could explain differences in their representation of the grassland bird assemblage studied. For example, the SWGSCA was originally established in areas of tallgrass prairie that was historically the dominant plant community (Wisconsin Department of Natural Resources, 2009). In contrast, the CWGCA is primarily managed for the greater prairie chicken (*Tympanuchus cupido*), another state‐threatened species (Hardy et al., 2020; Wisconsin Department of Natural Resources, 2004), and the WPHRA was primarily established for the conservation of both waterfowl and grassland birds (Wisconsin Department of Natural Resources, 2020). Unsurprisingly, we found SWGSCA to have the highest representation of grassland‐dependent birds among the three Focal Landscapes. These results suggest that conservation targets are concentrated in one Focal Landscape with the other areas having limited unique contributions to the conservation of the entire assemblage. Our findings highlight that the origins of the different conservation areas and their goals for targeting specific surrogates could have implications for the representation of other habitat specialist species in these conservation networks.

Citizen science has emerged worldwide (Dickinson et al., 2010) with initiatives targeting terrestrial and marine organisms (Norman et al., 2017; Sullivan et al., 2014). A major benefit of structured citizen science initiatives, such as breeding bird atlases, is the use of the standardized protocol in the collection of species‐rich data (La Sorte et al., 2018). These types of programs allow for direct comparisons across species with different ecological requirements and life histories and are an important tool in multitargeted evaluations of conservation planning. In this study, we found the existing conservation areas to have a higher representation for species that prefer intermediate‐height vegetation. This pattern can be explained by shortgrass specialists having some of the lowest sensitivity to urban and forested areas in our models (i.e., horned lark and vesper sparrow, Appendix S2), which would result in density estimates to be widespread across the state rather than concentrated in grassland hot spots. The sedge wren, a tallgrass specialist, was not represented by either Focal Landscapes or GBCA ensembles relative to the rest of the state. This can be explained by its association with wet prairie and idle planted grasslands (Elliott & Johnson, 2018) and its sensitivity to agricultural management practices that reduce late successional patches of dense herbaceous and woody vegetation that sedge wren use for foraging and cover from predators (Marx et al., 2008). Overall, our findings highlight the need for spatial planning of grassland conservation networks to account for heterogeneous habitats and integrate multispecies assessments (Elliott & Johnson, 2017; Nocera et al., 2007; Thogmartin et al., 2006).

Predictions of species occupancy or abundance are essential for informing conservation planning, but caution is required in the interpretation of these models. For example, while a traditional approach is to assign a common scale of effect across multiple species and covariates (i.e., Sólymos et al., 2020), our approach does not account for species–environment associations occurring across a diversity of scales (Jackson & Fahrig, 2012; Stuber & Fontaine, 2019). Among our sampled species, models also varied in their predictive performance. This variation may be due to inappropriate choice of predictors, spatial scales, or the need for targeted surveys for more rarely observed species, such as Henslow's sparrow and western meadowlark. Future work could include a multispecies approach when estimating model coefficients of rare species (e.g., Gomez et al., 2018). Finally, our analysis did not account for the connectivity of grassland patches, which may be important in identifying landscapes that are large enough to accommodate viable populations of grassland birds.

Grassland birds are of national and international conservation concern given the pronounced declines in North America, Great Britain, and western Europe (Askins et al., 2007; McCracken, 2005), and the challenges of developing conservation networks within a mosaic of competing land uses and socioeconomic activities (Dallimer & Strange, 2015; Thogmartin et al., 2014). For example, in the alpine grasslands of the Qinghai–Tibetan Plateau, less than 30% of grassland biodiversity hot spots were found inside protected areas (Su et al., 2019). Landscape‐scale simulations can help guide and evaluate the placement of conservation areas with alternative planning scenarios (Cannon et al., 2020; Dorning et al., 2015). However, there have been few attempts at testing whether biodiversity metrics measured within existing conservation areas are distinct from the broader landscape (but see Thornton et al., 2016 for an example). A spatial randomization approach is useful in determining whether the placement of conservation networks are effective at protecting unique or uncommon ecological features (Thornton et al., 2016). Here, we implemented a spatial randomization approach that has the distinct feature of generating replicates with the same shape and size of the conservation areas of interest. By maintaining the same spatial structure between replicate and actual conservation areas, significant differences between the two types of conservation areas are less likely to be detected, thus reducing the likelihood of falsely rejecting the null hypothesis (Beale et al., 2008). Given the projected changes in grassland land cover in this region (Lark, 2020), this spatial modeling approach can be useful in assessing shifts in conservation representation under future land use scenarios. This randomization approach can be further modified to identify candidate grassland landscapes for future grassland bird conservation efforts in Wisconsin. While this study focused on evaluating the representation within the existing grassland conservation network, future work could include other optimal spatial planning approaches that integrate additional aspects of conservation decision‐making such as land acquisition or restoration potential (e.g., Zonation or Marxan; Franklin et al., 2011; Kukkala & Moilanen, 2013) to inform potential expansions of the current conservation network.

## 5 CONCLUDING REMARKS

With only 4.6% of native temperate grassland being protected globally (Carbutt et al., 2017; Hoekstra et al., 2004), it is important to evaluate the overlap of these conservation areas with the distribution and abundance of declining grassland‐dependent taxa. The identification of potential conservation gaps could inform management plans and future expansions of existing conservation networks (Stem et al., 2005). In this study, we identified mismatches between an existing grassland conservation network and a rapidly declining assemblage of grassland‐dependent birds. While our findings provide important information to regional conservation initiatives, this study also outlines a flexible framework that combines large‐scale abundance data and spatial simulations with the potential to be applied to other conservation networks and serves as a complementary tool to conservation monitoring and spatial planning efforts globally. Large‐scale biodiversity databases and advancements in spatial tests, combined with habitat availability information and expert judgment, are key components of conservation efforts to halt and reverse the global trends of biodiversity loss.

## CONFLICT OF INTEREST

The authors have no conflicts to declare.

## AUTHOR CONTRIBUTION

**Laura A. Nunes:** Conceptualization (lead); Data curation (lead); Formal analysis (lead); Investigation (lead); Methodology (lead); Visualization (lead); Writing‐original draft (lead); Writing‐review & editing (lead). **Christine A. Ribic:** Conceptualization (equal); Funding acquisition (equal); Project administration (equal); Supervision (equal); Validation (equal); Writing‐review & editing (equal). **Benjamin Zuckerberg:** Conceptualization (equal); Funding acquisition (equal); Project administration (equal); Resources (equal); Software (equal); Supervision (equal); Validation (equal); Writing‐review & editing (equal).

### OPEN RESEARCH BADGES

This article has earned an Open Data badge for making publicly available the digitally‐shareable data necessary to reproduce the reported results. The data is available at https://doi.org/10.5061/dryad.cnp5hqc5p.

## Supporting information

Appendices S1‐S7

## Data Availability

The formatted data that support the findings of this study, including spatially explicit population density predictions, land cover, and climate measurements for Wisconsin and R code for conducting the RTR analysis, are available at Dryad: https://doi.org/10.5061/dryad.cnp5hqc5p.
