# Supplementary material for: Identifying mismatches between conservation area networks and vulnerable populations using spatial randomization
Source: Ecol Evol. 2021 Oct 25;11(22):16006–20. doi: 10.1002/ece3.8270 (PMC8601911; doi:10.1002/ece3.8270)
Supplement: Supplementary file 1 — Appendices S1‐S7 [file ECE3-11-16006-s001.docx]

[Supplementary Material 2](#_Toc81993264)

[**Appendix 1**: Further information on management goals in the three Focal Landscapes 2](#_Toc81993265)

[**Appendix 2**: Full description on modeling approach and results of model assessments 4](#_Toc81993266)

[Model goodness-of-fit and cross-validation 5](#_Toc81993267)

[Spearman’s rank correlations between predicted and observed density 14](#_Toc81993268)

[Model AUC analysis 15](#_Toc81993269)

[Spatial autocorrelation analysis 19](#_Toc81993270)

[Effect size of site covariates on abundance 21](#_Toc81993271)

[**Appendix 3:** Target statewide population analysis 22](#_Toc81993272)

[Optimal spatial planning map for model lower confidence intervals 22](#_Toc81993273)

[Optimal spatial planning map for model upper confidence intervals 23](#_Toc81993274)

[Proportion of statewide population predicted within existing conservation network based on model averages. 24](#_Toc81993275)

[Proportion of statewide population predicted within existing conservation network based on model lower confidence intervals. 24](#_Toc81993276)

[Proportion of statewide population predicted within existing conservation network based on model upper confidence intervals. 25](#_Toc81993277)

[**Appendix 4**: Full description of spatial randomization analysis using the RTR approach 27](#_Toc81993278)

[**Appendix 5**: Contemporary ecological baselines for 9 obligate grassland songbird species 30](#_Toc81993279)

[**Appendix 6:** Details on average species densities with confidence intervals 31](#_Toc81993280)

[**Appendix 7:** Spatial randomization analysis based on model confidence intervals 34](#_Toc81993281)

[Spatial randomization analysis with lower confidence intervals 34](#_Toc81993282)

[Spatial randomization analysis with upper confidence intervals 35](#_Toc81993283)

# Supplementary Material

## **Appendix 1**: Further information on management goals in the three Focal Landscapes

| Table S1: Full details on Focal Landscape and Grassland Bird Conservation Areas (GBCAs). | Management Goal | Established to conserve prairie remnants, open grasslands, savannas and important stream systems set within a working agricultural landscape (Wisconsin Department of Natural Resources 2009). Establishing and improving habitat within the 4 GBCAs. | Improving the GBCAs to maintain greater prairie-chicken populations and reducing the amount of tree/shrub cover in the Focal Landscape to enhance prairie-chicken movement among the GBCAs (Wisconsin Department of Natural Resources 2004). | Permanently protect and restore wetlands, grasslands, and savannas. Focus on the conservation of waterfowl and grassland birds (Wisconsin Department of Natural Resources 2020). |
| --- | --- | --- | --- | --- |
|  | Landscape Composition (Ribic et al. 2019) | Contains the largest amount of grassland (idle grasslands and pasture represent 37% of land use) and lowest amount of unsuitable habitat (developed and forest/shrubs represent 18% of land use). | Contains 22% grassland (idle grasslands and pasture) but the largest amount of unsuitable habitat (developed and forest/shrubs, 32% of land use). | Contains 23% grassland (idle grasslands and pasture) and 27% unsuitable habitat (developed and forest/shrubs) |
|  | Description | Encompasses numerous remnants of original tallgrass prairie and oak savannah. Given its proximity to urban centers, the area is experiencing rapid change as former farms are subdivided for residential housing. | State management focused on greater prairie-chicken (*Tympanuchus cupido*); Landscape remains in agricultural production; increasing tree and shrub cover in the landscape. | One-half of this landscape is in agriculture with another one-third in grasslands; dairy farming and row crops remain the predominant agricultural uses; the area is experiencing rapid urbanization along its western fringe. |
|  | Individual GBCAs | Barreltown,  Garrison Grove,  Military Ridge,  Perry Primrose | Buena Vista,  Leola,  Paul Olson | Rush River,  Star Prairie  Willow Rush |
|  | Focal Landscape | Southwest Grasslands and Stream Conservation Area (SWGSCA) | Central Wisconsin Grasslands Conservation Area (CWGCA) | Western Prairie Habitat Restoration Area (WPHRA) |

**
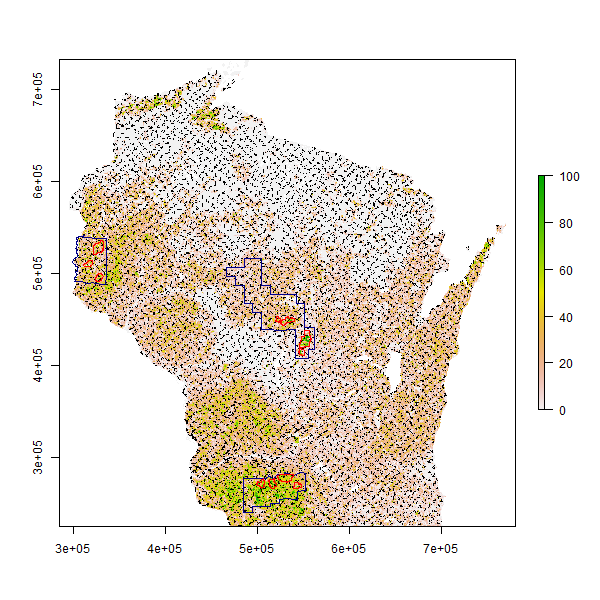
**

**Figure S1:** Percent of grassland cover within 1-km^2^ grid cells in Wisconsin (Wiscland 2.0). Focal Landscapes (purple) and Grassland Bird Conservation Areas (GBCAs; red) overlayed. Focal Landscapes refer to areas with high amounts of grasslands (i.e., grassy matrix) and low amounts of hostile habitat (i.e., urban, forest). GBCA ensembles refer to all Grassland Bird Conservation Areas within a Focal Landscape (i.e., smaller outlines within each Focal Landscape). CWGCA = Central Wisconsin Grasslands Conservation Area; SWGSCA = Southwest Grasslands and Stream Conservation Area; WPHRA = Western Prairie Habitat Restoration Area. Black dots refer to point count sites of Wisconsin’s Breeding Bird Atlas II

## **Appendix 2**: Full description on modeling approach and results of model assessments

Observers were selected for conducting point counts using a regional bird certification test (http://www.birdercertification.org) to ensure the observers were skilled at auditory and visual observations of Wisconsin birds. We obtained land cover from Wiscland 2.0, which is a Wisconsin dataset that utilized aerial imagery from 2010–2014 to classify land-cover into 8 generalized categories at a 30-m spatial resolution (Wisconsin Department of Natural Resources 2016). We obtained monthly climate data for May, June and July (i.e., mean, minimum, maximum temperature and total precipitation) from PRISM 30-year normals at 800-m resolution (Daly et al. 2008). We calculated proportion of each land cover class excluding water bodies (i.e., agriculture, barren, forest, grassland, shrubland, urban and wetland) and mean climate (i.e., mean, minimum and maximum temperature and average total precipitation) within a 100m buffer around each WBBA survey site using the ‘landscapemetrics’ R package (Hesselbarth et al. 2019); this scale approximates the maximum average territory size of grassland birds (1-3 ha) and is less than half the minimum distance between sampling sites in this study (Sólymos et al. 2020). To estimate statewide covariates at a 1-km^2^ resolution, the state of Wisconsin was divided into 1-km^2^ pixels, resulting in a total of 134,295 1-km^2^ pixels. We calculated proportion of land cover and measured mean climate within each 1-km^2^ pixel. We tested for multicollinearity among variables using Pearson’s correlation and variance inflation factor analysis (VIF). We removed minimum and mean temperatures because of higher correlations and lower VIF values with the other variables.

We generated species-specific site density estimates based on WBBA point-count data and *N*-mixture removal multinomial Poisson hierarchical models that account for imperfect detection (Royle 2004; Dorazio et al. 2005). We modeled site density using proportion of four land cover classes (urbanization, agriculture, grassland, and forest) and two climate variables (average maximum temperature and total precipitation of 30-year normal between May and July). The detection component included the annual Julian date that the surveys were conducted. We standardized all predictors by subtracting their means and dividing by the standard deviation. Models were fitted with observations from the 0-50 m distance bin to reduce observation uncertainty at longer distances (Diefenbach et al. 2003). The frequentist fitted model also included an area offset of 100 m to estimate population density at any spatial scale. We assessed model fit based on chi-square goodness-of-fit tests and overdispersion estimates using the function *Nmix.gof.test* and we corrected models with overdispersion >1 using the function *summaryOD* from the AICmodavg R package (Mazerolle 2016). We tested for the presence of spatial autocorrelation in model residuals using the *spline.correlog* function in the ncf R package to calculate Moran’s I between survey sites within a maximum distance of 30 km and using 100 resamples for the bootstrapping (Bjornstad & Bjornstad, 2016).

To predict population densities at a meaningful spatial scale for conservation planning (Sólymos et al., 2020), the state of Wisconsin was divided into 1-km^2^ pixels, resulting in a total of 134,295 1-km^2^ pixels. Proportion of the same 4 land cover categories and mean climate within each pixel were measured and fitted using the hierarchical model estimates to generate spatially-explicit predictions of population density for each 1-km^2^ pixel in Wisconsin. We predicted population densities using the average coefficients, and to account for error propagation we generated predictions using +/-1.96*SE of the model coefficients and conducted the analysis outlined below using the three sets of estimates (i.e., average, 2.5% and 97.5% confidence intervals).

| Table S2. Number of sites and individuals detected in 0-50m distance bands from Wisconsin’s Breeding Bird Atlas II | | |
| --- | --- | --- |
| Species | Number of sites detected | Total number of individuals detected |
| Bobolink (BOBO) | 207 | 268 |
| Dickcissel (DICK) | 410 | 486 |
| Eastern meadowlark (EAME) | 172 | 184 |
| Grasshopper sparrow (GRSP) | 49 | 55 |
| Henslow’s sparrow (HESP) | 22 | 26 |
| Horned lark (HOLA) | 352 | 408 |
| Savannah sparrow (SAVS) | 814 | 1014 |
| Sedge wren (SEWR) | 238 | 296 |
| Vesper sparrow (VESP) | 136 | 139 |
| Western meadowlark (WEME) | 12 | 12 |

### Model goodness-of-fit and cross-validation

**Bobolink (BOBO) ______________________________________________________________**

**Global model:**

multinomPois(formula = ~Date ~ urban + grass + ag + forest +

tmax + ppt + offset(log(area)), data = spFrame, control = list(maxit = 10000,

trace = TRUE, REPORT = 1))

Abundance (log-scale):

Estimate SE z P(>|z|)

(Intercept) -6.610 0.1567 -42.18 0.00e+00

urban -0.597 0.2000 -2.99 2.82e-03

grass 0.822 0.0988 8.32 9.05e-17

ag 0.418 0.1400 2.98 2.85e-03

forest -0.923 0.2302 -4.01 6.04e-05

tmax -0.503 0.0902 -5.58 2.47e-08

ppt 0.204 0.0821 2.48 1.31e-02

Detection (logit-scale):

Estimate SE z P(>|z|)

(Intercept) 0.4532 0.132 3.439 0.000584

Date -0.0952 0.130 -0.734 0.463084

AIC: 2802.803

Number of sites: 16542

optim convergence code: 0

optim iterations: 156

Bootstrap iterations: 0

**Goodness-of-fit test:**

Observed chi-square statistic = 147761.5

Number of bootstrap samples = 1000

P-value = 0.039

Quantiles of bootstrapped statistics:

0% 25% 50% 75% 100%

40403 61821 72898 91305 571219

Estimate of c-hat = 1.78

**Cross-validation:**

mean sd

RMSE 0.073463356 0.0083714722

MAE 0.006255201 0.0002917628

**Dickcissel (DICK) ______________________________________________________________**

**Global model:**

multinomPois(formula = ~Date ~ urban + grass + ag + forest +

tmax + ppt + offset(log(area)), data = spFrame, control = list(maxit = 10000,

trace = TRUE, REPORT = 1))

Abundance (log-scale):

Estimate SE z P(>|z|)

(Intercept) -6.401 0.1565 -40.89 0.00e+00

urban -0.204 0.1613 -1.27 2.06e-01

grass 0.792 0.1127 7.02 2.17e-12

ag 0.914 0.1518 6.02 1.76e-09

forest -0.934 0.2522 -3.70 2.12e-04

tmax -0.237 0.0880 -2.70 7.02e-03

ppt 0.556 0.0632 8.78 1.57e-18

Detection (logit-scale):

Estimate SE z P(>|z|)

(Intercept) 1.3378 0.102 13.162 1.45e-39

Date 0.0513 0.108 0.477 6.33e-01

AIC: 4115.344

Number of sites: 16542

optim convergence code: 0

optim iterations: 198

Bootstrap iterations: 0

**Goodness-of-fit test:**

Observed chi-square statistic = 313090.1

Number of bootstrap samples = 1000

P-value = 0.019

Quantiles of bootstrapped statistics:

0% 25% 50% 75% 100%

28692 48915 60096 81317 1433955

Estimate of c-hat = 3.83

**Cross-validation:**

mean sd

RMSE 0.08158236 0.004655576

MAE 0.01075683 0.000390936

**Eastern Meadowlark (EAME) ___________________________________________________**

**Global model:**

multinomPois(formula = ~Date ~ urban + grass + ag + forest +

tmax + ppt + offset(log(area)), data = spFrame, control = list(maxit = 10000,

trace = TRUE, REPORT = 1))

Abundance (log-scale):

Estimate SE z P(>|z|)

(Intercept) -7.57148 0.296 -25.612 1.12e-144

urban -0.00862 0.169 -0.051 9.59e-01

grass 0.75630 0.133 5.699 1.20e-08

ag 0.20790 0.187 1.114 2.65e-01

forest -1.71941 0.390 -4.413 1.02e-05

tmax -0.26331 0.132 -1.996 4.59e-02

ppt 0.52666 0.100 5.247 1.55e-07

Detection (logit-scale):

Estimate SE z P(>|z|)

(Intercept) 0.4639 0.141 3.279 0.00104

Date 0.0691 0.138 0.503 0.61530

AIC: 1980.717

Number of sites: 16542

optim convergence code: 0

optim iterations: 177

Bootstrap iterations: 0

**Goodness-of-fit test:**

Observed chi-square statistic = 91013.43

Number of bootstrap samples = 1000

P-value = 0.169

Quantiles of bootstrapped statistics:

0% 25% 50% 75% 100%

28642 48224 60545 78705 1654533

Estimate of c-hat = 1.18

**Cross-validation:**

mean sd

RMSE 0.047539387 0.003544373

MAE 0.004163143 0.000138968

**Grasshopper sparrow (GRSP) ___________________________________________________**

**Global model:**

multinomPois(formula = ~Date ~ urban + grass + ag + forest +

tmax + ppt + offset(log(area)), data = spFrame, control = list(maxit = 10000,

trace = TRUE, REPORT = 1))

Abundance (log-scale):

Estimate SE z P(>|z|)

(Intercept) -8.386 0.440 -19.04 7.80e-81

urban 1.250 1.000 1.25 2.11e-01

grass 2.337 0.908 2.57 1.00e-02

ag 2.330 1.240 1.88 6.03e-02

forest 2.151 1.562 1.38 1.68e-01

tmax 0.773 0.284 2.72 6.56e-03

ppt -0.442 0.200 -2.21 2.72e-02

Detection (logit-scale):

Estimate SE z P(>|z|)

(Intercept) 0.32975 0.245 1.3458 0.178

Date 0.00476 0.236 0.0201 0.984

AIC: 764.6367

Number of sites: 16542

optim convergence code: 0

optim iterations: 212

Bootstrap iterations: 0

**Goodness-of-fit test:**

Observed chi-square statistic = 118019.3

Number of bootstrap samples = 1000

P-value = 0.07

Quantiles of bootstrapped statistics:

0% 25% 50% 75% 100%

18500 44013 56516 76494 405456

Estimate of c-hat = 1.75

**Cross-validation:**

mean sd

RMSE 0.029896933 0.0069564254

MAE 0.001362341 0.0001468544

**Henslow’s sparrow (HESP) ______________________________________________________**

**Global model:**

multinomPois(formula = ~Date ~ urban + grass + ag + forest +

tmax + ppt + offset(log(area)), data = spFrame, control = list(maxit = 10000,

trace = TRUE, REPORT = 1))

Abundance (log-scale):

Estimate SE z P(>|z|)

(Intercept) -9.8084 0.783 -12.525 5.47e-36

urban -1.6177 1.059 -1.527 1.27e-01

grass 0.6278 0.289 2.172 2.99e-02

ag -0.0330 0.403 -0.082 9.35e-01

forest -1.5078 0.821 -1.836 6.64e-02

tmax 0.9636 0.451 2.138 3.25e-02

ppt -0.0516 0.293 -0.176 8.60e-01

Detection (logit-scale):

Estimate SE z P(>|z|)

(Intercept) 0.32 0.453 0.706 0.480402

Date -1.72 0.511 -3.362 0.000773

AIC: 381.2058

Number of sites: 16542

optim convergence code: 0

optim iterations: 189

Bootstrap iterations: 0

**Goodness-of-fit test:**

Observed chi-square statistic = 27508.06

Number of bootstrap samples = 1000

P-value = 0.509

Quantiles of bootstrapped statistics:

0% 25% 50% 75% 100%

6651 19351 27664 41591 4245318

Estimate of c-hat = 0.53

**Cross-validation:**

mean sd

RMSE 0.0168341283 0.0062918588

MAE 0.0006080849 0.0001229533

**Horned lark (HOLA) ___________________________________________________________**

**Global model:**

multinomPois(formula = ~Date ~ urban + grass + ag + forest +

tmax + ppt + offset(log(area)), data = spFrame, control = list(maxit = 10000,

trace = TRUE, REPORT = 1))

Abundance (log-scale):

Estimate SE z P(>|z|)

(Intercept) -8.8190 0.4925 -17.907 1.05e-71

urban 1.3540 0.5937 2.280 2.26e-02

grass 2.0145 0.5404 3.728 1.93e-04

ag 3.3114 0.7346 4.508 6.55e-06

forest 0.2094 1.0300 0.203 8.39e-01

tmax -0.0695 0.1035 -0.671 5.02e-01

ppt 0.5280 0.0706 7.475 7.72e-14

Detection (logit-scale):

Estimate SE z P(>|z|)

(Intercept) 0.199 0.110 1.81 0.070170

Date -0.424 0.117 -3.63 0.000285

AIC: 3625.047

Number of sites: 16542

optim convergence code: 0

optim iterations: 173

Bootstrap iterations: 0

**Goodness-of-fit test:**

Observed chi-square statistic = 99203.77

Number of bootstrap samples = 1000

P-value = 0.08

Quantiles of bootstrapped statistics:

0% 25% 50% 75% 100%

22985 33914 40518 54837 1647698

Estimate of c-hat = 1.64

**Cross-validation:**

mean sd

RMSE 0.076104935 0.0042353709

MAE 0.009362146 0.0002813372

**Savannah sparrow** (SAVS)**______________________________________________________**

**Global model:**

multinomPois(formula = ~Date ~ urban + grass + ag + forest +

tmax + ppt + offset(log(area)), data = spFrame, control = list(maxit = 10000,

trace = TRUE, REPORT = 1))

Abundance (log-scale):

Estimate SE z P(>|z|)

(Intercept) -5.291 0.0840 -62.956 0.00e+00

urban 0.369 0.1122 3.292 9.96e-04

grass 1.153 0.0926 12.458 1.26e-35

ag 1.405 0.1260 11.147 7.37e-29

forest -0.123 0.1812 -0.678 4.97e-01

tmax -0.707 0.0481 -14.689 7.63e-49

ppt 0.314 0.0429 7.323 2.42e-13

Detection (logit-scale):

Estimate SE z P(>|z|)

(Intercept) 0.71633 0.0588 12.176 4.15e-34

Date -0.00737 0.0572 -0.129 8.97e-01

AIC: 7870.518

Number of sites: 16542

optim convergence code: 0

optim iterations: 125

Bootstrap iterations: 0

**Goodness-of-fit test:**

Observed chi-square statistic = 99138.79

Number of bootstrap samples = 1000

P-value = 0.17

Quantiles of bootstrapped statistics:

0% 25% 50% 75% 100%

48414 68089 77536 92884 258545

Estimate of c-hat = 1.19

**Cross-validation:**

mean sd

RMSE 0.12222184 0.0037411195

MAE 0.02216859 0.0005027759

**Sedge wren (SEWR) ___________________________________________________________**

**Global model:**

multinomPois(formula = ~Date ~ urban + grass + ag + forest +

tmax + ppt + offset(log(area)), data = spFrame, control = list(maxit = 10000,

trace = TRUE, REPORT = 1))

Abundance (log-scale):

Estimate SE z P(>|z|)

(Intercept) -6.1808 0.1236 -49.987 0.00e+00

urban -1.4204 0.1618 -8.779 1.65e-18

grass -0.2711 0.0477 -5.681 1.34e-08

ag -0.8476 0.0655 -12.949 2.39e-38

forest -2.0226 0.1253 -16.147 1.20e-58

tmax 0.0900 0.0872 1.032 3.02e-01

ppt -0.0271 0.0817 -0.331 7.40e-01

Detection (logit-scale):

Estimate SE z P(>|z|)

(Intercept) 0.8881 0.114 7.806 5.92e-15

Date -0.0877 0.110 -0.797 4.26e-01

AIC: 3041.394

Number of sites: 16542

optim convergence code: 0

optim iterations: 179

Bootstrap iterations: 0

**Goodness-of-fit test:**

Observed chi-square statistic = 127129.6

Number of bootstrap samples = 1000

P-value = 0.067

Quantiles of bootstrapped statistics:

0% 25% 50% 75% 100%

39107 58886 69375 86358 508773

Estimate of c-hat = 1.58

**Cross-validation:**

mean sd

RMSE 0.065620481 0.004120303

MAE 0.006825912 0.000204767

**Vesper sparrow (VESP)_________________________________________________________**

**Global model:**

multinomPois(formula = ~Date ~ urban + grass + ag + forest +

tmax + ppt + offset(log(area)), data = spFrame, control = list(maxit = 10000,

trace = TRUE, REPORT = 1))

Abundance (log-scale):

Estimate SE z P(>|z|)

(Intercept) -6.814 0.182 -37.45 5.15e-307

urban 0.721 0.383 1.88 5.96e-02

grass 1.006 0.348 2.89 3.86e-03

ag 1.807 0.464 3.90 9.71e-05

forest 1.306 0.592 2.21 2.73e-02

tmax 0.811 0.185 4.39 1.12e-05

ppt -0.308 0.135 -2.28 2.29e-02

Detection (logit-scale):

Estimate SE z P(>|z|)

(Intercept) 0.220 0.157 1.41 0.159

Date 0.191 0.152 1.26 0.209

AIC: 1754.901

Number of sites: 16542

optim convergence code: 0

optim iterations: 172

Bootstrap iterations: 0

**Goodness-of-fit test:**

Observed chi-square statistic = 98440.2

Number of bootstrap samples = 1000

P-value = 0.154

Quantiles of bootstrapped statistics:

0% 25% 50% 75% 100%

41630 63725 73417 88219 426884

Estimate of c-hat = 1.21

**Cross-validation:**

mean sd

RMSE 0.040550 1.831707e-03

MAE 0.003311 9.986855e-05

**Western meadowlark (WEME) __________________________________________________**

**Global model:**

multinomPois(formula = ~Date ~ urban + grass + ag + forest +

tmax + ppt + offset(log(area)), data = spFrame, control = list(maxit = 10000,

trace = TRUE, REPORT = 1))

Abundance (log-scale):

Estimate SE z P(>|z|)

(Intercept) -9.4710 0.552 -17.1510 6.18e-66

urban 0.0154 0.817 0.0188 9.85e-01

grass 0.8493 0.590 1.4386 1.50e-01

ag 0.6962 0.825 0.8441 3.99e-01

forest -0.1439 1.101 -0.1308 8.96e-01

tmax -1.1212 0.424 -2.6467 8.13e-03

ppt 1.1700 0.406 2.8834 3.93e-03

Detection (logit-scale):

Estimate SE z P(>|z|)

(Intercept) 27.1 NaN NaN NaN

Date 6.3 NaN NaN NaN

AIC: 251.9492

Number of sites: 16542

optim convergence code: 0

optim iterations: 234

Bootstrap iterations: 0

**Goodness-of-fit test:**

Observed chi-square statistic = 9.125817e+15

Number of bootstrap samples = 1000

P-value = 0

Quantiles of bootstrapped statistics:

0% 25% 50% 75% 100%

1275 7177 10341 13588 88879

Estimate of c-hat = 810114390547

**Cross-validation:**

mean sd

RMSE 0.0122200680 2.427851e-03

MAE 0.0002964182 3.652702e-05

### Spearman’s rho between predicted and observed density values.

| **Table S3.** Results Spearman’s rho (r_s_) between observed and predicted density values using the full dataset. | | |
| --- | --- | --- |
| Species name | Specie code | r_s_ |
| Grasshopper sparrow | GRSP | 0. 0682 |
| Horned lark | HOLA | 0. 2089 |
| Vesper sparrow | VESP | 0. 1016 |
| Western meadowlark | WEME | 0. 0337 |
| Bobolink | BOBO | 0. 1405 |
| Dickcissel | DICK | 0. 2023 |
| Eastern meadowlark | EAME | 0. 1408 |
| Savannah sparrow | SAVS | 0. 2772 |
| Henslow’s sparrow | HESP | 0. 0503 |
| Sedge wren | SEWR | 0. 1417 |

### Model AUC analysis


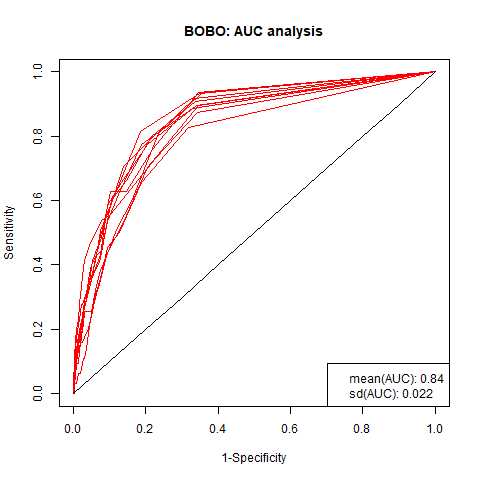

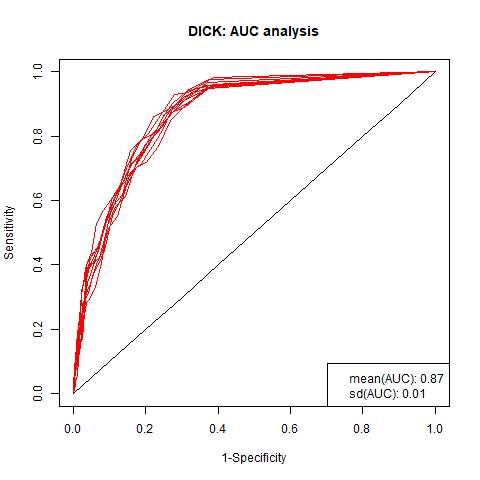

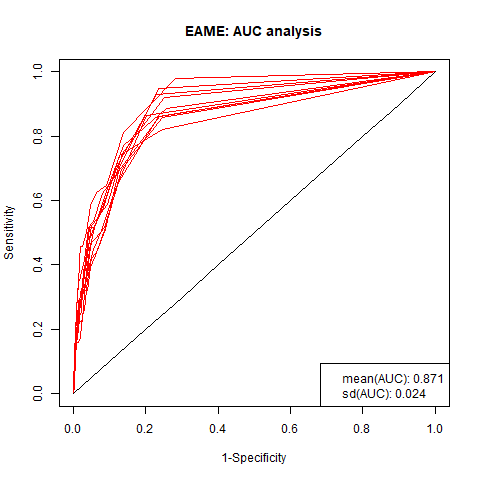

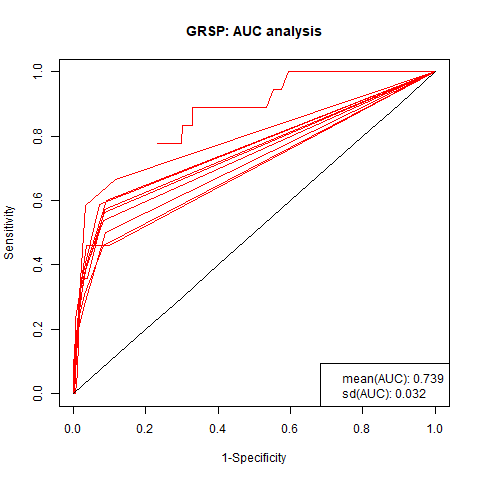

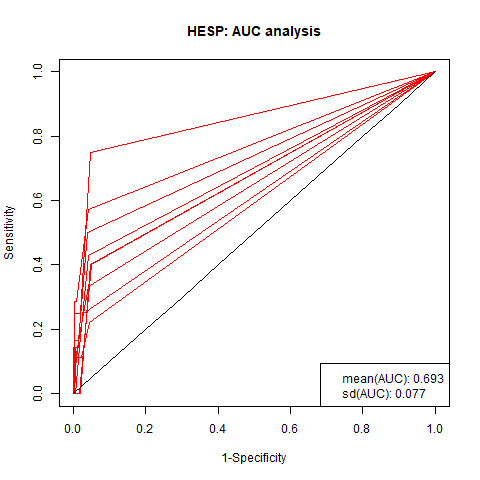

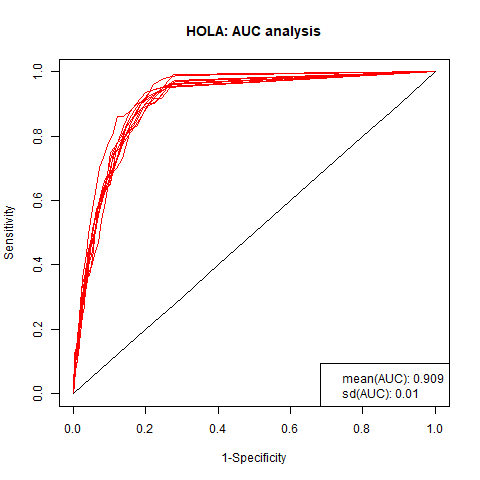

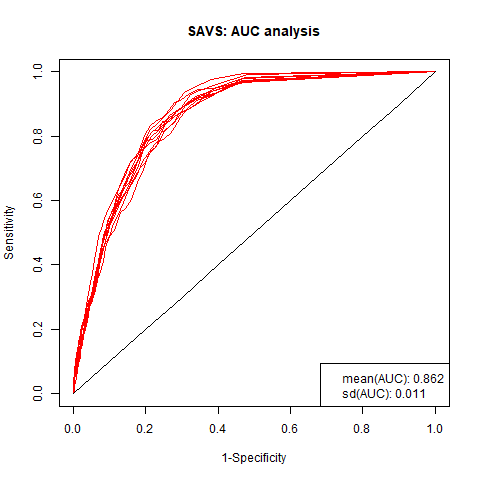

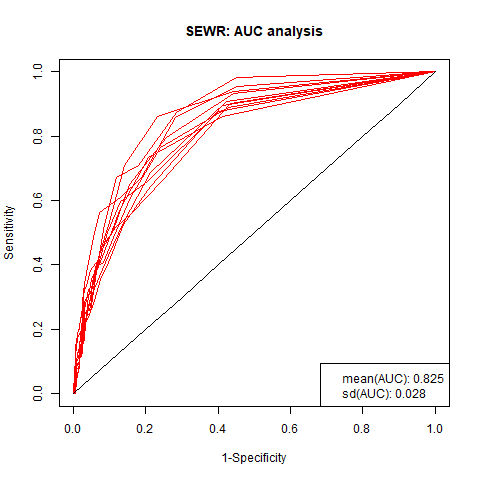

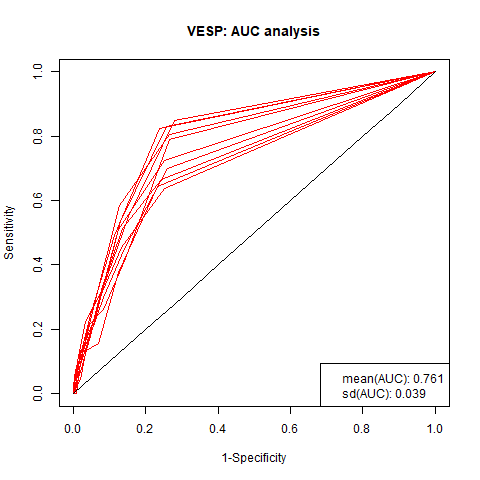


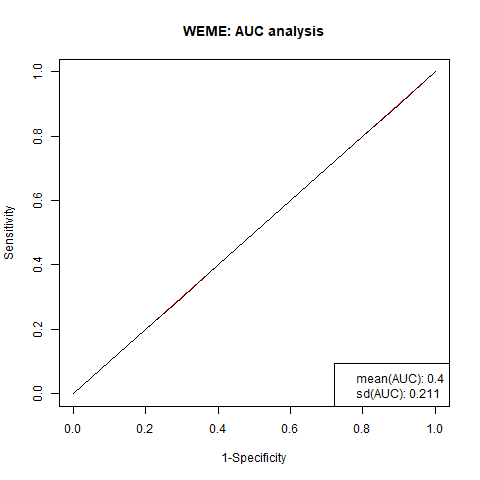


### Spatial autocorrelation analysis

*R code:*

*model.res<-apply(residuals(sp.model),1,mean) ##mean residuals per site*

*mod<- ncf:: spline.correlog(x = sites.xy[, c("x")], y = sites.xy[, ("y")], z = mod.res, resamp = 100, xmax = 30000)*

*plot(spline.res,main=paste0(sp.code,': Spline correlogram'),xlab='Distance m')*


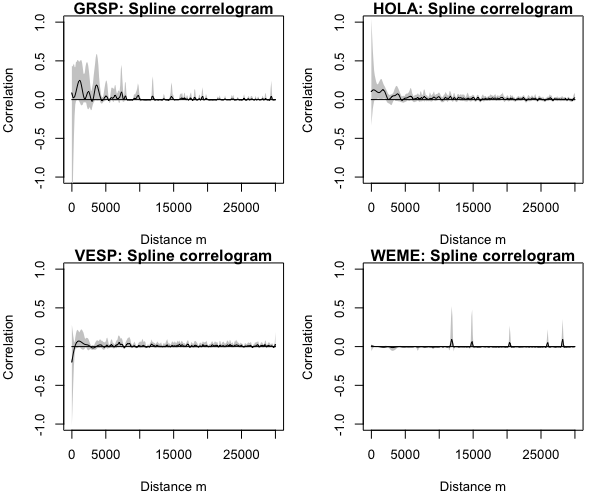


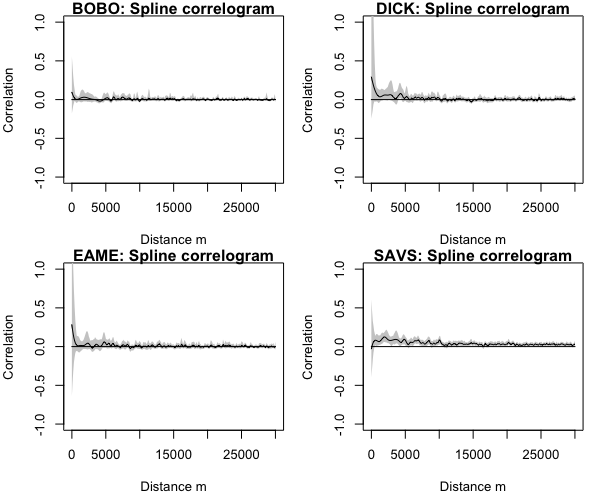


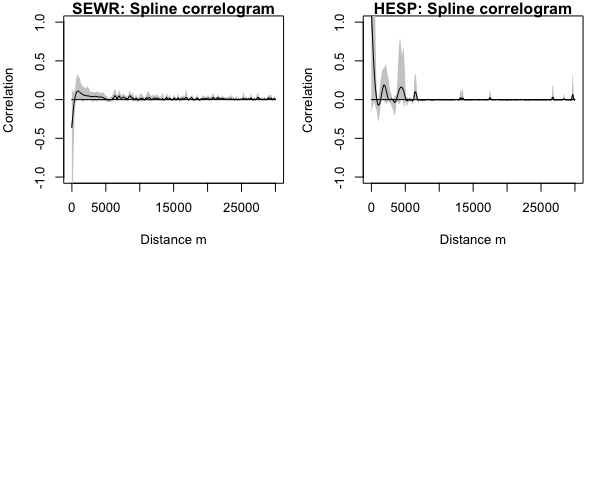


| **Table S4.** Results from spline correlogram to test for spatial autocorrelation up to 30km distance | | | |
| --- | --- | --- | --- |
| Species name | Species code | Estimate | Quantile (0.025 ; 0.975) |
| Grasshopper sparrow | GRSP | 0.09 | (-1.79 ; 0.44) |
| Horned lark | HOLA | 0.10 | (-0.33 ; 1.04) |
| Vesper sparrow | VESP | -0.20 | (-1.02 ; 0.28) |
| Western meadowlark | WEME | 0.01 | (-0.06 ; 0.03) |
| Bobolink | BOBO | 0.09 | (-0.18 ; 0.56) |
| Dickcissel | DICK | 0.29 | (-0.25 ; 2.52) |
| Eastern meadowlark | EAME | 0.29 | (-0.64 ; 1.67) |
| Savannah sparrow | SAVS | -0.03 | (-0.39 ; 0.61) |
| Sedge wren | SEWR | -0.36 | (-1.74 ; 0.15) |
| Henslow’s sparrow | HESP | 1.18 | (-0.17 ; 9.08) |

### Effect size of site covariates on abundance


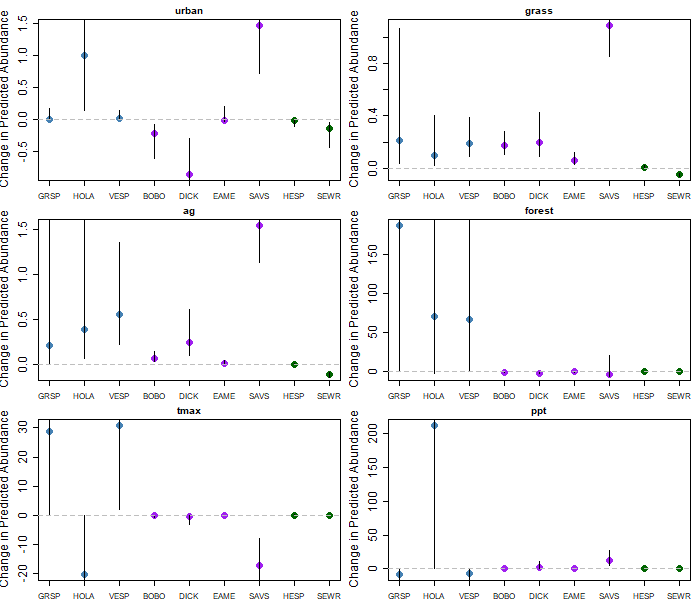


## **Appendix 3:** Target statewide population analysis

### Optimal spatial planning map for model lower confidence intervals


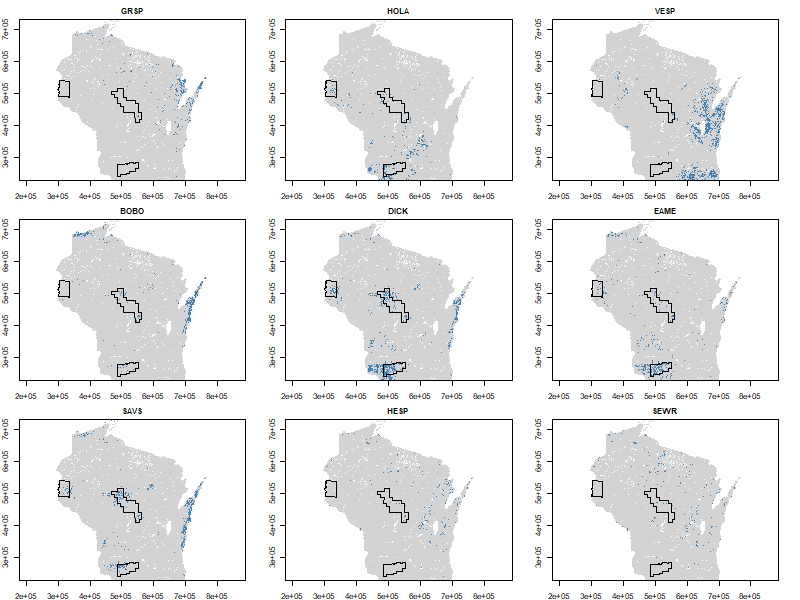


**Figure S2.** Optimal spatial planning for 9 obligate grassland songbirds grouped by habitat specialism (blue for shortgrass specialists, purple for midgrass specialists and green for tallgrass specialists). Blue pixels represent the smallest set of 1-km^2^ grid cells that were predicted to encompass at least 20% of the statewide population. Outlines of existing Focal Landscapes are overlayed in black. Abundance predictions based on model lower confidence intervals.

### Optimal spatial planning map for model upper confidence intervals


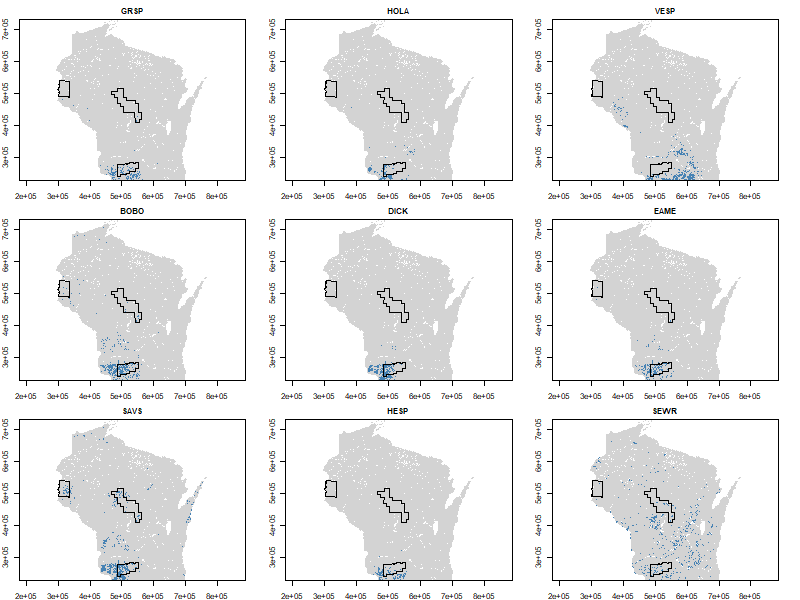


**Figure S3.** Optimal spatial planning for 9 obligate grassland songbirds grouped by habitat specialism (blue for shortgrass specialists, purple for midgrass specialists and green for tallgrass specialists). Blue pixels represent the smallest set of 1-km^2^ grid cells that were predicted to encompass at least 20% of the statewide population. Outlines of existing Focal Landscapes are overlayed in black. Abundance predictions based on model upper confidence intervals.

### Proportion of statewide population predicted within existing conservation network based on model averages.

| Table S5. Total predicted statewide abundances, and minimum number of grid cells to meet 20% of population targets compared to the area of the existing conservation network and the statewide population predicted within the conservation network. Analysis based on model coefficient averages. | | | | | |
| --- | --- | --- | --- | --- | --- |
|  | Predicted statewide abundance of (breeding males) | 20% statewide abundance | Minimum set of grid cells that includes 20% of statewide population  (x 1km^2^) | Area of existing conservation network  ( x 1km^2^) | statewide population within conservation network  (breeding males) |
| BOBO | 42347 | 8469 | 4261 | 7240 | 5391 |
| DICK | 90257 | 18051 | 3057 | 7240 | 13363 |
| EAME | 24587 | 4917 | 2097 | 7240 | 4146 |
| GRSP | 7865 | 1573 | 3856 | 7240 | 869 |
| HESP | 4519 | 904 | 2714 | 7240 | 544 |
| HOLA | 67598 | 13520 | 1767 | 7240 | 9110 |
| SAVS | 175526 | 35105 | 4287 | 7240 | 23048 |
| SEWR | 74844 | 14969 | 3159 | 7240 | 4455 |
| VESP | 33643 | 6729 | 4835 | 7240 | 2191 |

### Proportion of statewide population predicted within existing conservation network based on model lower confidence intervals.

| Table S6. Total predicted statewide abundances, and minimum number of grid cells to meet 20% of population targets compared to the area of the existing conservation network and the statewide population predicted within the conservation network. Analysis based on model coefficient lower confidence intervals (CI). | | | | | |
| --- | --- | --- | --- | --- | --- |
|  | Predicted statewide abundance of (breeding males) | 20% statewide abundance | Minimum set of grid cells that includes 20% of statewide population  (x 1km^2^) | Area of existing conservation network  ( x 1km^2^) | statewide population within conservation network  (breeding males) |
| BOBO | 37454 | 7491 | 2493 | 7240 | 3219 |
| DICK | 47267 | 9453 | 5152 | 7240 | 5661 |
| EAME | 14138 | 2828 | 3273 | 7240 | 1793 |
| GRSP | 4806 | 961 | 986 | 7240 | 108 |
| HESP | 2487 | 497 | 1693 | 7240 | 138 |
| HOLA | 10823 | 2165 | 2858 | 7240 | 1319 |
| SAVS | 147484 | 29497 | 3818 | 7240 | 17442 |
| SEWR | 80344 | 16069 | 1863 | 7240 | 3469 |
| VESP | 17050 | 3410 | 6367 | 7240 | 939 |


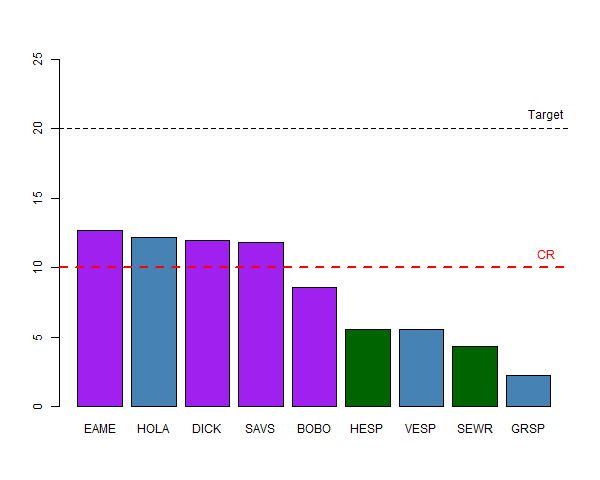


Figure S4. Percentage of statewide population within existing Focal Landscapes. Colors distinguish between habitat specialists (blue for shortgrass specialists, purple for midgrass specialists and green for tallgrass specialists). Refer to Table S2 for species abbreviations. Dotted black line represented the conservation target of 20% of statewide population to be within existing conservation areas (i.e., Focal Landscapes). Dotted red line represents the 10% threshold for IUCN’s critical endangered status (Johnston et al., 2020). Analysis based on model lower confidence intervals.

### Proportion of statewide population predicted within existing conservation network based on model upper confidence intervals.

| Table S7. Total predicted statewide abundances, and minimum number of grid cells to meet 20% of population targets compared to the area of the existing conservation network and the statewide population predicted within the conservation network. Analysis based on model coefficient upper confidence intervals (CI). | | | | | |
| --- | --- | --- | --- | --- | --- |
|  | Predicted statewide abundance of (breeding males) | 20% statewide abundance | Minimum set of grid cells that includes 20% of statewide population  (x 1km^2^) | Area of existing conservation network  ( x 1km^2^) | statewide population within conservation network  (breeding males) |
| BOBO | 66246 | 13249 | 2969 | 7240 | 10262 |
| DICK | 234795 | 46959 | 1974 | 7240 | 37566 |
| EAME | 57726 | 11545 | 1370 | 7240 | 10853 |
| GRSP | 198686 | 39737 | 1238 | 7240 | 26372 |
| HESP | 37754 | 7551 | 1049 | 7240 | 4858 |
| HOLA | 527730 | 105546 | 1216 | 7240 | 74170 |
| SAVS | 220029 | 44006 | 4109 | 7240 | 31201 |
| SEWR | 86197 | 17239 | 4649 | 7240 | 6465 |
| VESP | 96183 | 19237 | 3236 | 7240 | 6580 |


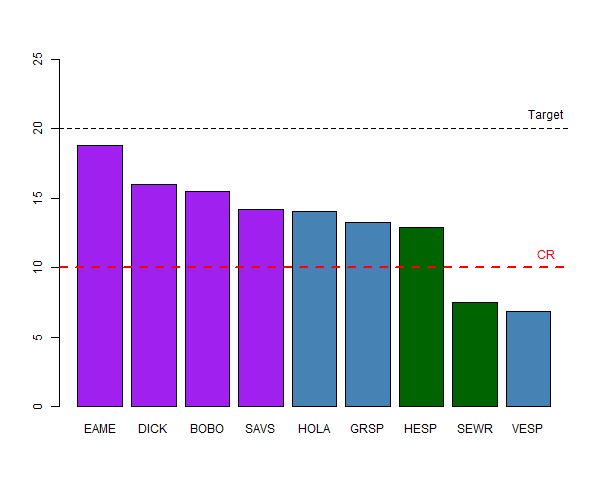


Figure S5. Percentage of statewide population within existing Focal Landscapes. Colors distinguish between habitat specialists (blue for shortgrass specialists, purple for midgrass specialists and green for tallgrass specialists). Refer to Table S2 for species abbreviations. Dotted black line represented the conservation target of 20% of statewide population to be within existing conservation areas (i.e., Focal Landscapes). Dotted red line represents the 10% threshold for IUCN’s critical endangered status (Johnston et al., 2020). Analysis based on model upper confidence intervals.

## **Appendix 4**: Full description of spatial randomization analysis using the RTR approach

To determine the current distributions of grassland birds in the state, we identified density hotspots for individual species using a top percentage approach (Orme et al. 2005; Wilson et al. 2019) that consisted of using pixels with density values within the top 10% of all pixels of each species statewide density. Then, we measured the percentage of individual species density hotspots found within the existing conservation network (i.e., all Focal Landscapes combined) (Su et al. 2019).

To determine how the Focal Landscapes and GBCAs compare to other potentially suitable grassland areas across the state, we used a randomization, or spatial null model, approach (Gotelli 2001). This null model approach allows us to compare the densities of grassland songbirds within the existing conservation network with potential densities captured by a conservation planning approach that is uninformed by political boundaries, expert opinion, or biodiversity information. First, we calculated contemporary population baselines as the mean density estimates of all 1-km^2^ grid cells within each Focal Landscape or GBCA. We used a 500-m buffer around the landscape boundaries to ensure that all grid cells intersecting with the landscape boundaries were included. We then tested whether the placement of Focal Landscapes and GBCAs was “optimal” for conserving species density hotspots in our suite of grassland birds. We defined an existing conservation area of interest (i.e., Focal Landscapes or GBCA ensemble) to be optimal for the conservation of a given species when the corresponding population baseline density was higher than population densities from replicate areas with a random spatial arrangement (i.e., null replicates, (Gotelli 2001; Nunes & Pearson 2017).

We adapted the Random-Translation-Rotation (RTR) model of Nunes & Pearson (2017) to generate replicates that maintained the same size and shape of the conservation areas of interest. In short, we placed replicates randomly within a sampling region (i.e., the state of Wisconsin or within a Focal Landscape; Figure S1). This approach generated a distribution of 1000 possible population densities based on unconstrained placement of individual conservation areas across the state. To test whether baseline densities were significantly higher than expected by random chance, we contrasted the actual population baseline densities against their corresponding null distributions. Population baseline densities within either Focal Landscapes or GBCA ensembles were determined to be significantly different from other suitable grassland areas when the population densities of the landscape or ensemble were greater than 90% of the densities of replicate areas.

To constrain the random placement of replicates to suitable grassland areas, we used a three-step process: 1) We defined any 1-km^2^ grid cell that had at least 50% of total unsuitable land cover (i.e., forest and urban land types) as unsuitable for grassland bird conservation; 2) we calculated the average amount of unsuitable grassland habitat within all conservation areas studied (i.e., Focal Landscapes and GBCA ensembles) based on Wiscland 2.0 (see above); 3) We discarded any null replicates that encompassed more than 20% of unsuitable grassland bird habitat (average amount of unsuitable habitat among the studied conservation areas) and replaced them with replicates that fell in areas with <20% of unsuitable habitat, thus ensuring comparability with current conservation areas (Figure S2).


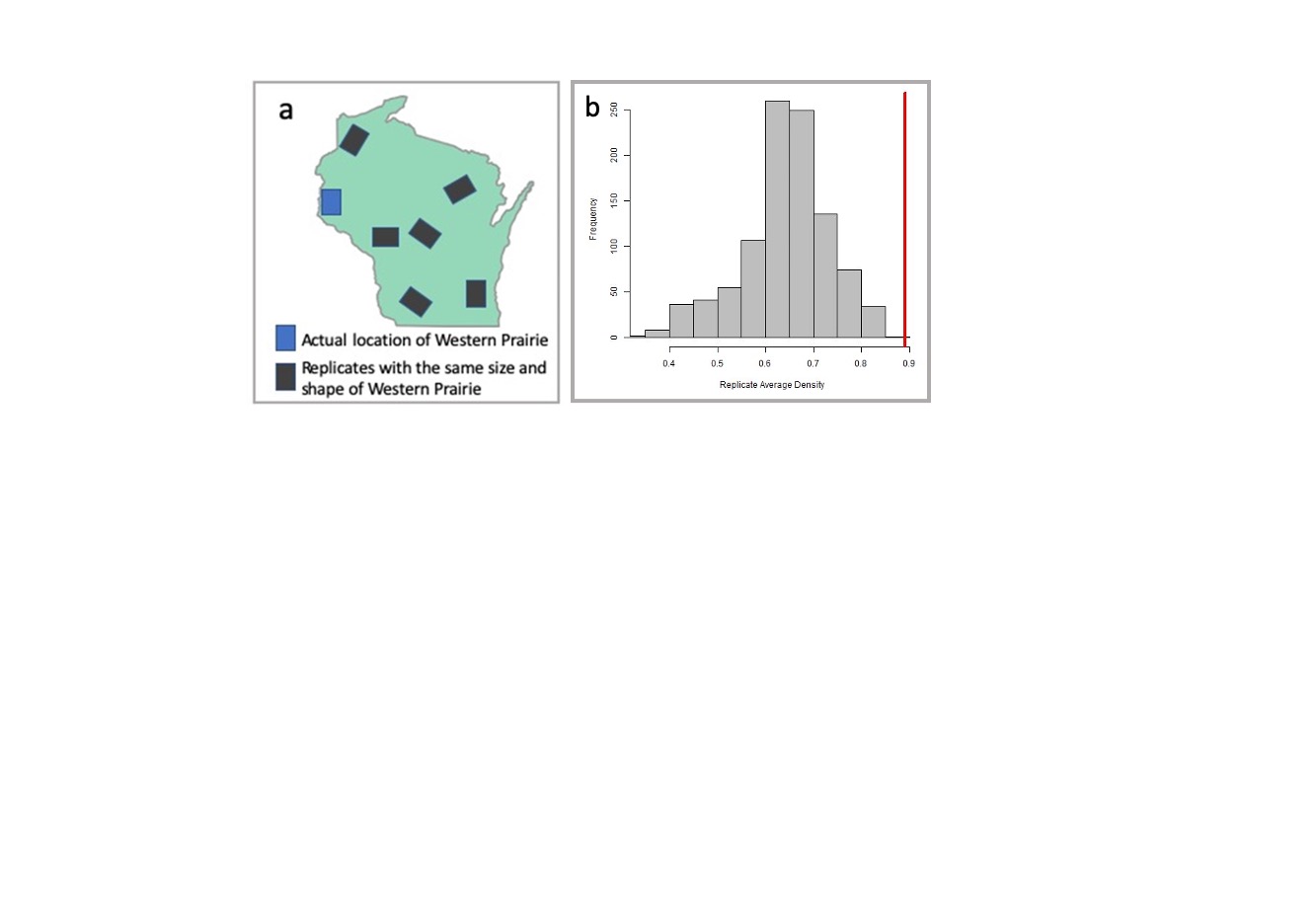


Figure S6. Conceptual representation of the Random translation and rotation (RTR) null model applied to the Western Prairie (adapted from Nunes and Pearson, 2017). a) Polygons with the same shape and size of Western Prairies are randomly rotated and translated onto the Wisconsin landscape but constrained to only areas with dominant grassland habitat. b) We estimate average density at each replicate site and compile a null distribution of average density values (grey) from which we can contrast the observed densities (red) found in the Western Prairie Focal Landscape.


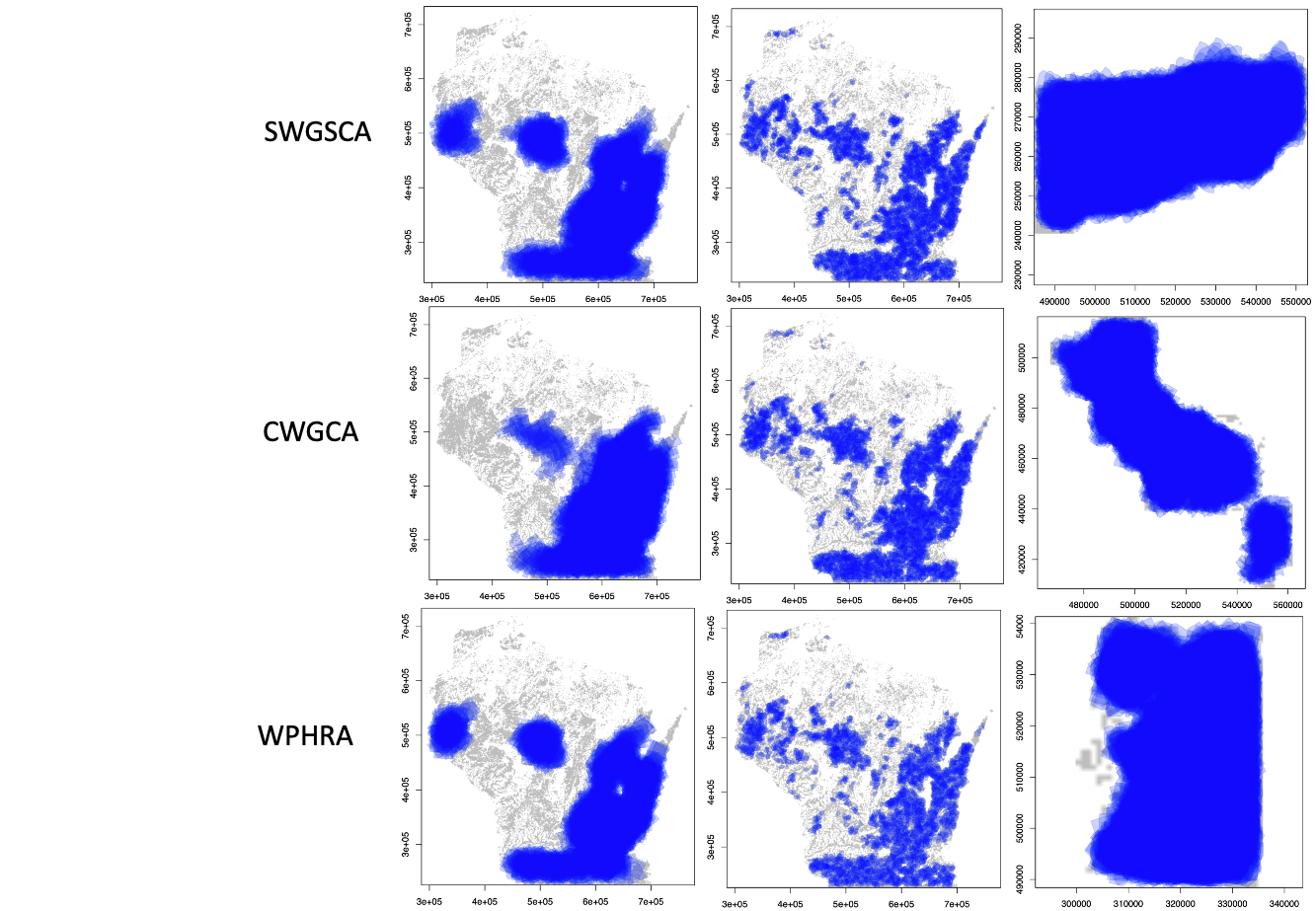


Figure S7. Replicate random landscapes (blue) for “Focal Landscape vs Statewide” scenario (left) and “GBCA vs Statewide” scenario (right) for each Focal Landscape and GBCA ensemble. Grey represents suitable grassland habitat and white represents unsuitable grassland habitat (i.e., more than 50% of forest or urban land cover).

## **Appendix 5**: Contemporary ecological baselines for 9 obligate grassland songbird species


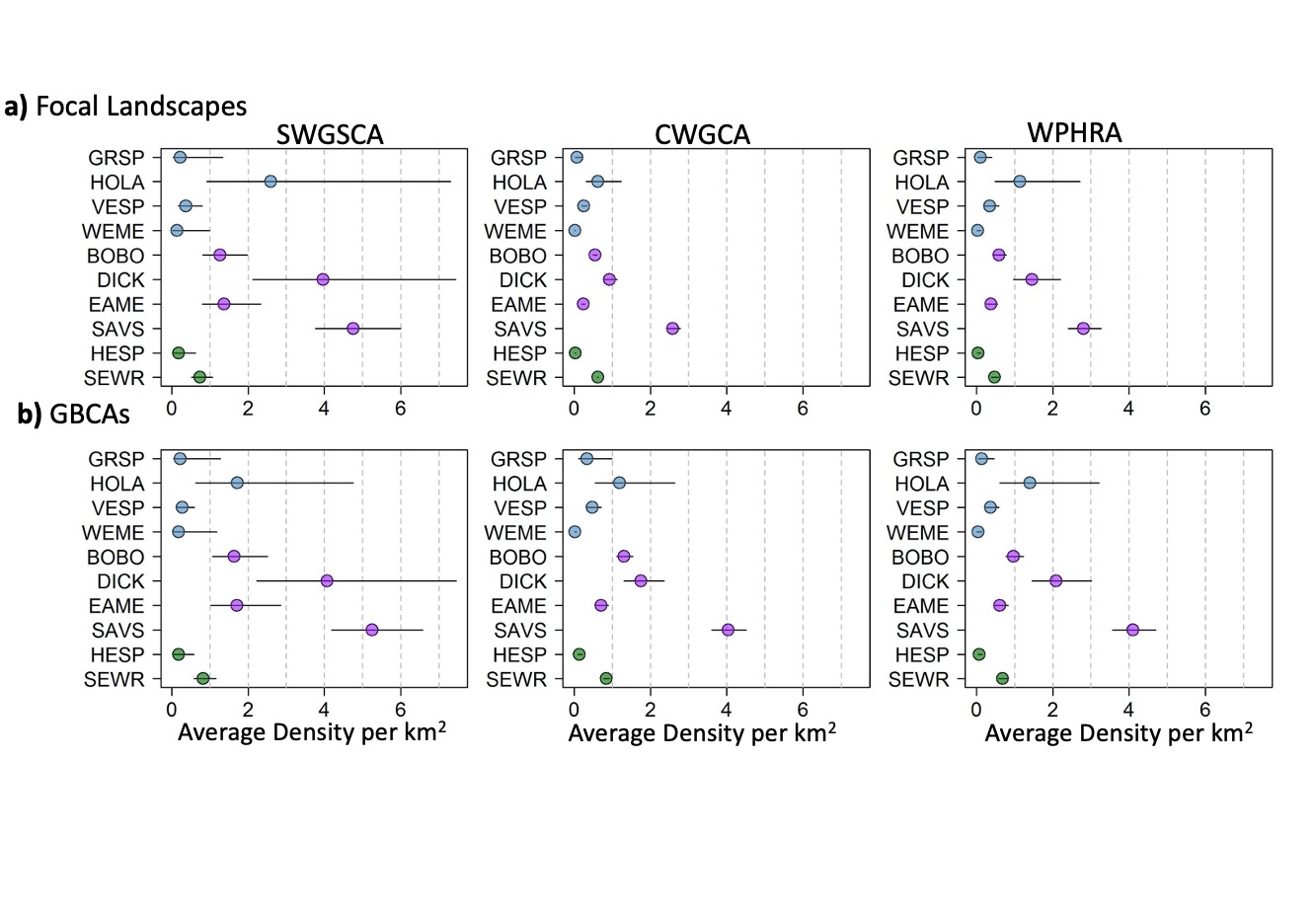


Figure S8. Contemporary population baselines for 9 obligate grassland songbird species, as the average population density within (a) individual Focal Landscapes and (b) GBCA ensembles. Circles represent the average population densities based on model coefficient averages and solid black lines represent average densities based on 2.5% and 97.5% confidence intervals (mean +/- 1.96* standard errors) of model coefficients. Sample sizes for Focal Landscapes are SWGSCA: 1945 x 1km^2^; CWGCA: 3670 x 1km^2^; WPHRA: 1631 x 1km^2^. Sample sizes for GBCAs are SWGSCA: 108 x 1km^2^; CWGCA: 154 x 1km^2^; WPHRA: 53 x 1km^2^.

## **Appendix 6:** Details on average species densities with confidence intervals

Table S8. Estimated Population Densities of breeding males in the state of Wisconsin. Average population densities based on model coefficient averages, and 2.5% and 97.5% confidence based on mean coefficients +/- 1.96*standard error (SE) of the model. Sample size = 140780 x 1km^2^

| Species Code | Average breeding males per km^2^ based on average model coefficients | Average breeding males per km^2^ based on lower confidence interval of model coefficients  (mean – 1.96*SE) | Average breeding males per km^2^ based on upper confidence interval of model coefficients  (mean + 1.96*SE) |
| --- | --- | --- | --- |
| BOBO | 0.30 | 0.27 | 0.47 |
| DICK | 0.64 | 0.34 | 1.67 |
| EAME | 0.17 | 0.10 | 0.41 |
| GRSP | 0.06 | 0.03 | 1.41 |
| HESP | 0.03 | 0.02 | 0.27 |
| HOLA | 0.48 | 0.08 | 3.75 |
| SAVS | 1.25 | 1.05 | 1.56 |
| SEWR | 0.53 | 0.57 | 0.61 |
| VESP | 0.24 | 0.12 | 0.68 |

Table S9. Estimated Population Densities of breeding males in Wisconsin’s Focal Landscapes. Average population densities based on model coefficient averages, and 2.5% and 97.5% confidence based on mean coefficients +/- 1.96*standard error (SE) of the model.

| Focal Landscape | Species Code | Average breeding males per km^2^ based on average model coefficients | Average breeding males per km^2^ based on lower confidence interval of model coefficients  (mean – 1.96*SE) | Average breeding males per km^2^ based on upper confidence interval of model coefficients  (mean + 1.96*SE) |
| --- | --- | --- | --- | --- |
| SWGSCA |  |  |  |  |
| (sample size= 1945 x 1km^2^) | BOBO | 1.27 | 0.51 | 3.16 |
|  | DICK | 3.96 | 1.14 | 14.00 |
|  | EAME | 1.37 | 0.45 | 4.24 |
|  | GRSP | 0.22 | 0.01 | 10.70 |
|  | HESP | 0.18 | 0.02 | 2.12 |
|  | HOLA | 2.59 | 0.26 | 26.30 |
|  | SAVS | 4.76 | 3.04 | 7.45 |
|  | SEWR | 0.74 | 0.37 | 1.52 |
|  | VESP | 0.37 | 0.08 | 1.72 |
| CWGCA |  |  |  |  |
| (sample size= 3670 x 1km^2^) | BOBO | 0.55 | 0.46 | 0.68 |
|  | DICK | 0.93 | 0.67 | 1.39 |
|  | EAME | 0.24 | 0.17 | 0.36 |
|  | GRSP | 0.08 | 0.02 | 0.65 |
|  | HESP | 0.03 | 0.02 | 0.07 |
|  | HOLA | 0.63 | 0.15 | 2.95 |
|  | SAVS | 2.59 | 2.27 | 2.98 |
|  | SEWR | 0.62 | 0.62 | 0.65 |
|  | VESP | 0.26 | 0.16 | 0.46 |
| WPHRA |  |  |  |  |
| (sample size= 1631 x 1km^2^) | BOBO | 0.59 | 0.34 | 1.05 |
|  | DICK | 1.46 | 0.66 | 3.38 |
|  | EAME | 0.38 | 0.18 | 0.84 |
|  | GRSP | 0.10 | 0.01 | 2.07 |
|  | HESP | 0.05 | 0.01 | 0.31 |
|  | HOLA | 1.15 | 0.17 | 7.88 |
|  | SAVS | 2.80 | 2.10 | 3.77 |
|  | SEWR | 0.48 | 0.32 | 0.72 |
|  | VESP | 0.34 | 0.13 | 1.00 |

Table S10. Estimated Population Densities of breeding males in Wisconsin’s Grassland Bird Conservation Area ensembles (GBCAs). Average population densities based on model coefficient averages, and 2.5% and 97.5% confidence based on mean coefficients +/- 1.96*standard error (SE) of the model.

| GBCA | Species Code | Average breeding males per km^2^ based on average model coefficients | Average breeding males per km^2^ based on lower confidence interval of model coefficients  (mean – 1.96*SE) | Average breeding males per km^2^ based on upper confidence interval of model coefficients  (mean + 1.96*SE) |
| --- | --- | --- | --- | --- |
| SWGSCA |  |  |  |  |
| (sample size= 108 x 1km^2^) | BOBO | 1.64 | 0.68 | 3.92 |
|  | DICK | 4.07 | 1.22 | 13.65 |
|  | EAME | 1.71 | 0.58 | 5.08 |
|  | GRSP | 0.23 | 0.01 | 9.45 |
|  | HESP | 0.18 | 0.02 | 1.82 |
|  | HOLA | 1.72 | 0.18 | 16.64 |
|  | SAVS | 5.25 | 3.40 | 8.11 |
|  | SEWR | 0.82 | 0.42 | 1.63 |
|  | VESP | 0.28 | 0.06 | 1.22 |
| CWGCA |  |  |  |  |
| (sample size= 154 x 1km^2^) | BOBO | 1.31 | 0.96 | 1.82 |
|  | DICK | 1.76 | 0.98 | 3.20 |
|  | EAME | 0.71 | 0.43 | 1.18 |
|  | GRSP | 0.34 | 0.04 | 3.56 |
|  | HESP | 0.14 | 0.07 | 0.33 |
|  | HOLA | 1.20 | 0.21 | 7.02 |
|  | SAVS | 4.04 | 3.25 | 5.02 |
|  | SEWR | 0.85 | 0.70 | 1.03 |
|  | VESP | 0.48 | 0.23 | 1.04 |
| WPHRA |  |  |  |  |
| (sample size= 53 x 1km^2^) | BOBO | 0.98 | 0.61 | 1.58 |
|  | DICK | 2.09 | 1.02 | 4.38 |
|  | EAME | 0.61 | 0.32 | 1.19 |
|  | GRSP | 0.14 | 0.01 | 1.91 |
|  | HESP | 0.08 | 0.02 | 0.29 |
|  | HOLA | 1.40 | 0.23 | 9.04 |
|  | SAVS | 4.10 | 3.14 | 5.36 |
|  | SEWR | 0.69 | 0.49 | 0.96 |
|  | VESP | 0.37 | 0.15 | 0.93 |

## **Appendix 7:** Spatial randomization analysis based on model confidence intervals

### Spatial randomization analysis with lower confidence intervals


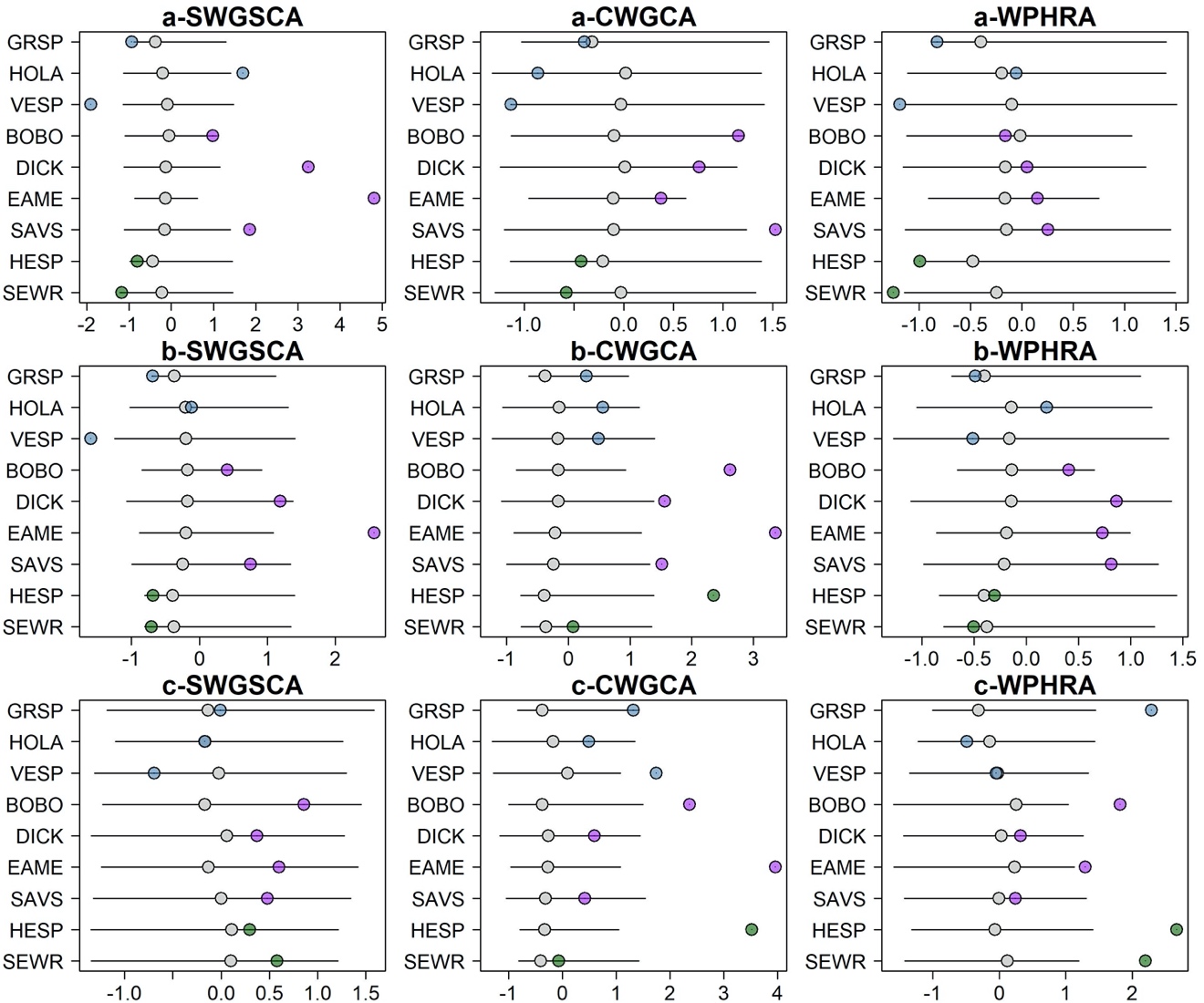


Figure S9. Results from RTR analysis on species statewide abundance using model estimates based on lower confidence intervals (i.e., mean - 1.96*SE). “Focal Landscape vs Statewide” Scenario (“a”-upper panels), “GBCAs vs Statewide” Scenario (“b”-middle panels) and “GBCAs vs Focal Landscape” Scenario (“c”-bottom panels). Analysis based on model lower confidence intervals.

### Spatial randomization analysis with upper confidence intervals


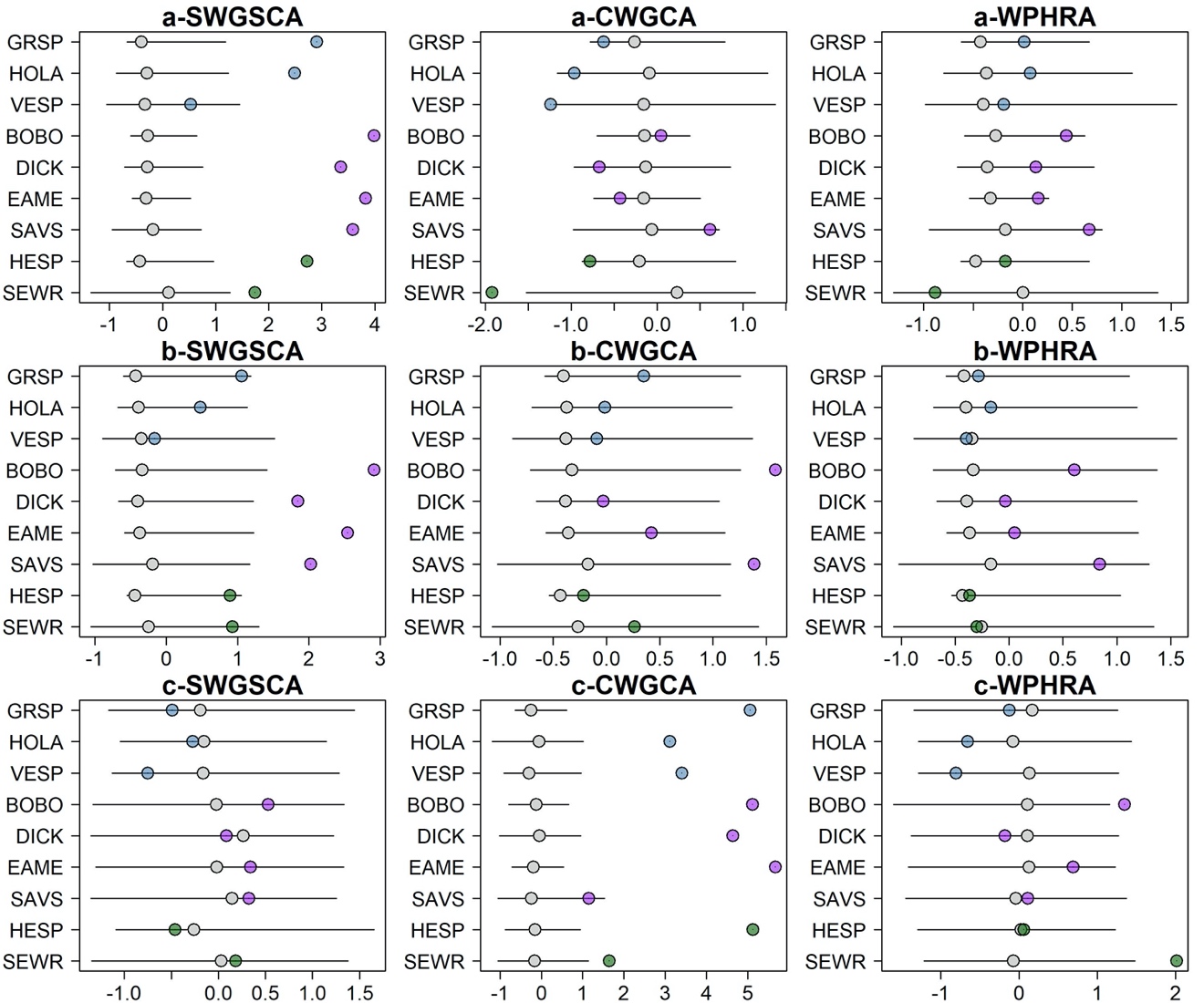


Figure S10. Results from RTR analysis on species statewide abundance using model estimates based on lower confidence intervals (i.e., mean - 1.96*SE). “Focal Landscape vs Statewide” Scenario (“a”-upper panels), “GBCAs vs Statewide” Scenario (“b”-middle panels) and “GBCAs vs Focal Landscape” Scenario (“c”-bottom panels). Analysis based on model upper confidence intervals.

**Literature Cited**

Bjornstad ON, Bjornstad MON. 2016. Package ‘ncf.’ Spatial nonparametric covariance functions. **1.1-7**

Diefenbach DR, Brauning DW, Mattice JA. 2003. Variability in grassland bird counts related to observer differences and species detection rates. The Auk **120**:1168–1179.

Dorazio RM, Jelks HL, Jordan F. 2005. Improving removal‐based estimates of abundance by sampling a population of spatially distinct subpopulations. Biometrics **61**:1093–1101.

Gotelli NJ. 2001. Research frontiers in null model analysis. Global ecology and biogeography **10**:337–343.

Mazerolle MJ. 2016. AICcmodavg: Model selection and multimodel inference based on (Q) AIC (c). R package version **1**.

Nunes LA, Pearson RG. 2017. A null biogeographical test for assessing ecological niche evolution. Journal of Biogeography **44**:1331–1343.

Orme CDL, Davies RG, Burgess M, Eigenbrod F, Pickup N, Olson VA, Webster AJ, Ding T-S, Rasmussen PC, Ridgely RS. 2005. Global hotspots of species richness are not congruent with endemism or threat. Nature **436**:1016–1019.

Ribic CA, Guttery, Michael R., Sample, David W., Schneider, Daniel, Paulios, Andy, Dadisman, John, Horton, Josephine A., Trosen, Christopher. 2019. Base data for three focal landscapes in Wisconsin used for grassland bird conservation. Forest Service Research Data Archive.https://doi.org/10.2737/RDS-2018-0065

Royle JA. 2004. N‐mixture models for estimating population size from spatially replicated counts. Biometrics **60**:108–115.

Su X, Han W, Liu G, Zhang Y, Lu H. 2019. Substantial gaps between the protection of biodiversity hotspots in alpine grasslands and the effectiveness of protected areas on the Qinghai-Tibetan Plateau, China. Agriculture, Ecosystems & Environment **278**:15–23.

Wilson S, Schuster R, Rodewald AD, Bennett JR, Smith AC, La Sorte FA, Verburg PH, Arcese P. 2019. Prioritize diversity or declining species? Trade-offs and synergies in spatial planning for the conservation of migratory birds in the face of land cover change. Biological Conservation **239**:108285. [Online]

Wisconsin Department of Natural Resources. 2004. Feasibility Study and Environmental Analysis for the Central Wisconsin Grassland Conservation Area. Wisconsin Department of Natural Resources. Available from https://p.widencdn.net/mrkdzl/cwgcafeas.

Wisconsin Department of Natural Resources. 2009. Feasibility Study, Master Plan and Environmental Impact Statement for the Southwest Wisconsin Grassland & Stream Conservation Area. Wisconsin Department of Natural Resources. Available from https://p.widencdn.net/y4rodd/FL_MP_SouthwestWisconsinGrassland_MasterPlan_2009.

Wisconsin Department of Natural Resources. 2020. Western Prairie Regional Master Plan: Master Plan and Environmental Analysis. Wisconsin Department of Natural Resources. Available from https://widnr.widen.net/view/pdf/tz1ghwjszz/FL_EL_WP_FinalMasterPlan.pdf.
